# Osteoarthritis: Mechanisms and Therapeutic Advances

**DOI:** 10.1002/mco2.70290

**Published:** 2025-08-01

**Authors:** Wei Liu, Ning‐Yi Guo, Jian‐Quan Wang, Bing‐Bing Xu

**Affiliations:** ^1^ Department of Sports Medicine Peking University Third Hospital, Institute of Sports Medicine of Peking University Beijing China; ^2^ Beijing Key Laboratory of Sports Injuries Beijing China; ^3^ Engineering Research Center of Sports Trauma Treatment Technology and Devices, Ministry of Education Beijing China

**Keywords:** chondrocyte, osteoarthritis (OA), pathogenesis, signaling pathway, therapy

## Abstract

Osteoarthritis (OA) is a chronic joint disease characterized by a complex pathological mechanism, including chondrocyte dysfunction, synovial inflammation, subchondral bone remodeling, and molecular regulation abnormalities. Key signaling pathways such as nuclear factor‐κB, mitoase‐activated protein kinase, and transforming growth factor‐β are disrupted, leading to cytokine imbalance, oxidative stress, and excessive protease activity, which collectively contribute to cartilage degeneration. This review summarizes the potential causes of OA, focusing on cellular and structural abnormalities in cartilage, synovial tissue, and subchondral bone, as well as dysregulation of signaling pathways, gene regulation, and molecular mechanisms. Given the limitations of current diagnostic methods for OA, biomarkers may offer new hope. Emerging therapeutic strategies for OA include biologics, intelligent drug delivery, and tissue engineering, aiming to modulate the immune microenvironment while promoting cartilage repair. However, these approaches face challenges such as long‐term safety and scalability. Future research may require deeper multidisciplinary collaboration and combination therapies to revolutionize the management of OA and improve patient outcomes.

## Graphical abstract

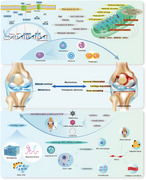

## 1 Introduction

**FIGURE 1:**
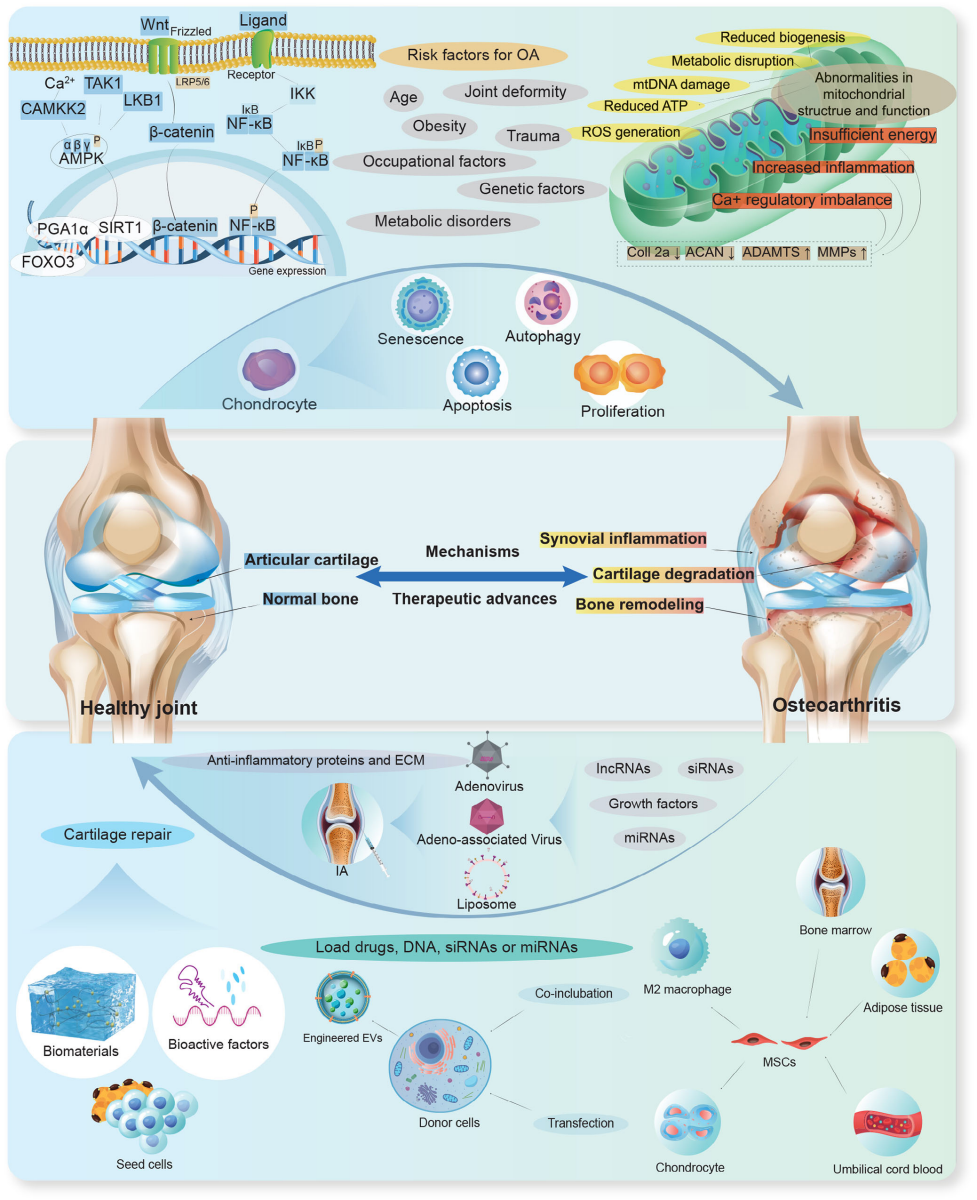
A schematic illustration of the pathogenesis and emerging therapeutic strategies for OA, including: risk factors and molecular mechanisms of OA, joint changes between healthy and OA conditions, and current emerging therapeutic approaches for OA. *Abbreviations*: IA: intra‐articular injection; EVs: extracellular vesicles.

Osteoarthritis (OA) is a chronic joint disease characterized by cartilage degeneration and calcification, synovial inflammation, and subchondral bone remodeling [1]. It is the most common joint degenerative disease, which often occurs middle‐aged and elderly individuals aged 45 years and older [2, 3]. OA patients mainly show joint pain, swelling, limited movement, and joint deformity [4], Long‐term disease leads to a serious decline in the quality of life, which may induce cardiovascular and psychological diseases [5, 6]. Multiple factors are known to increase the risk of OA, including genetics, body weight, and age [7] (Figure 1). OA can also be secondary to trauma, infection, and joint instability [7]. With the increase of obesity and exercise in recent years, OA patients have become younger [8]. By 2050, the global number of people living with knee OA is expected to reach 642 million [9, 10], resulting in huge healthcare costs and social burden [11, 12].

**FIGURE 2:**
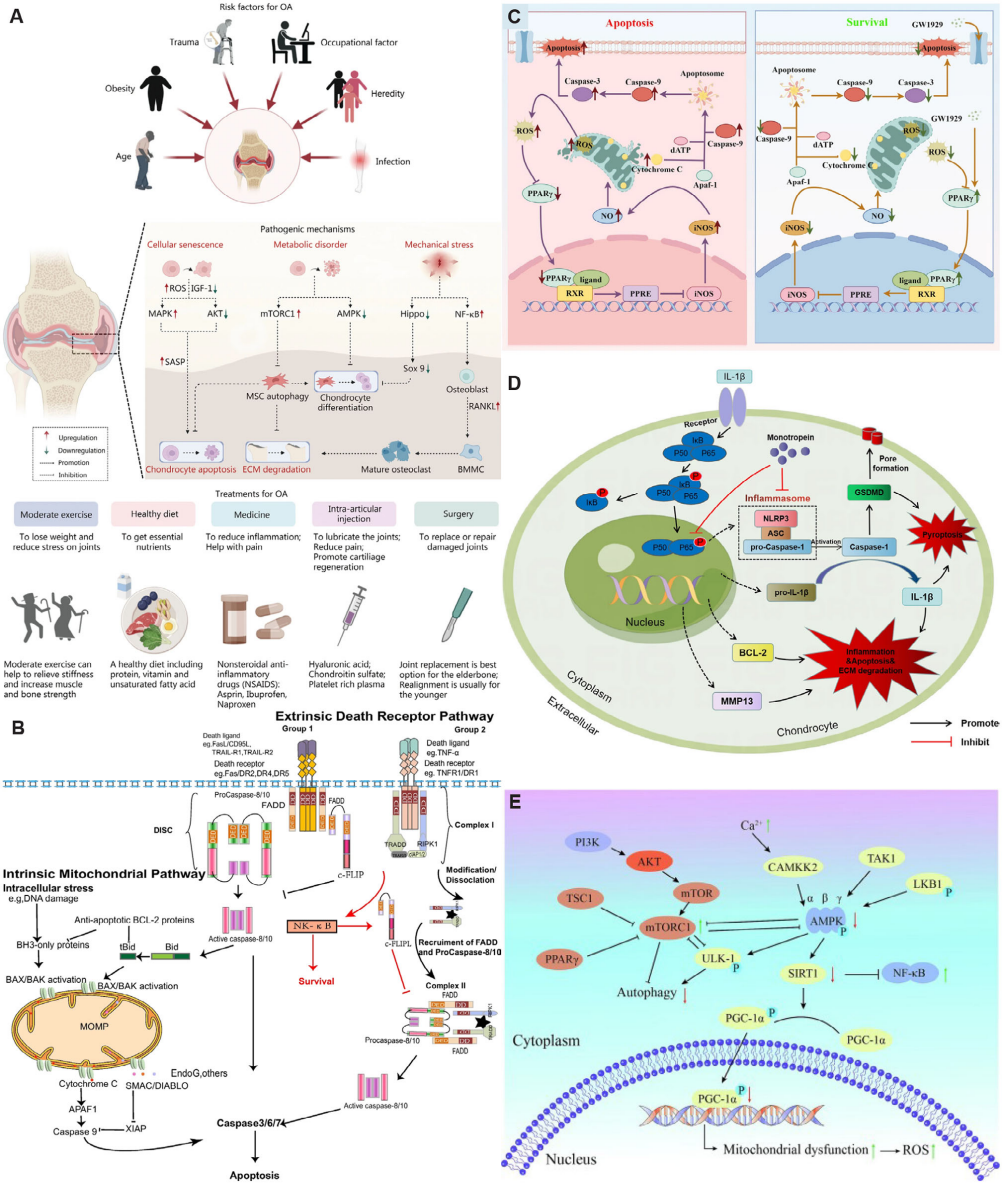
Cellular senescence and apoptosis mechanisms in the progression of osteoarthritis. (A) Risk factors, pathogenesis, and common treatments for OA. Reproduced with permission from Ref. [50], Copyright 2023, Springer Nature. (B) There are two pathways of apoptosis, the extrinsic and intrinsic pathways. The extrinsic (death receptor) apoptosis pathway involves the binding of death receptor ligands to members of the death receptor family (members of the tumor necrosis receptor superfamily) and intracellular signaling, which activates the intrinsic (also known as the mitochondrial) apoptosis pathway. Reproduced with permission from Ref. [25], Copyright 2023, Springer Nature. (C) PPARγ downregulates the production of inducible nitric oxide synthase, inhibits the generation of ROS, maintains the stability of MMPs, and blocks the activation of caspase‐9 and caspase‐3. Reproduced with permission from Ref. [27], Copyright 2024, Springer Nature. (D) Monotropein inhibits the degradation of the cartilage matrix and apoptosis of chondrocytes in both in vitro and in vivo models of osteoarthritis by targeting the NF‐κB signaling pathway. Reproduced with permission from Ref. [33], Copyright 2023, Springer Nature. (E) The autophagy and metabolism of chondrocytes are regulated by the AMPK and mTOR signaling pathways. Reproduced with permission from Ref. [38], Copyright 2023, Elsevier.

Since the specific pathogenesis of OA is still unclear [7], current treatments mainly focus on relieving joint pain and delaying the progress of OA, including drug therapy, physical therapy, and surgical treatment [13, 14] (Figure 2A). In the early OA, symptoms are relieved by proper exercise, weight loss, and nonsteroidal anti‐inflammatory drugs (NSAIDs) [15]. Patients with late‐stage OA are often accompanied by severe articular cartilage wear, and the injection of hyaluronic acid (HA) or hormone can only relieve the short‐term symptoms [16]. Eventually, patients require surgical treatment for joint replacement [17]. However, almost all of these treatments cannot fundamentally change the pathological state, causing great obstacles to the patient's life activities [18].

We reviewed the current understanding of the pathogenesis of OA, focusing on the interactions and cellular biology among cartilage, synovium, and subchondral bone. We explored the roles and functions of pathogenic signaling pathways and key molecules in OA, along with their associated clinical implications. Additionally, we analyzed the limitations of existing diagnostic methods and the emergence of novel biomarkers. Finally, we reviewed emerging therapeutic strategies for OA and clinical trials currently applied to OA patients.

## 2 The Pathogenesis of OA

Maintaining normal joint physiological function requires an intact anatomical structure. However, the progression of OA is frequently accompanied by alterations in the cellular and tissue architecture within the joint. Chondrocytes exhibit excessive apoptosis, abnormal autophagy, cellular senescence, and functional dysregulation, leading to an imbalance in the synthesis and degradation of the extracellular matrix (ECM) of cartilage. Synovial hyperplasia, fibrosis, angiogenesis, and inflammatory cell infiltration contribute to increased joint inflammation. Concurrently, dysregulated activity of osteoclasts and osteoblasts in the subchondral bone further disrupts joint stress distribution. These changes drive the progression of OA.

### 2.1 The Normal Anatomical and Physiological Structural Basis of the Joints

Joint is an important structure connecting the bone, including the ball and socket joint (hip joint), hinge joint (knee joint), and other types [19], these joints have some common basic institutions and physiological characteristics, and this review has mainly focused on knee OA. The knee joint consists mainly of bony structures and soft tissue accessory structures [20]. Bone structures include the distal femur, proximal tibia, and patella; and soft tissue accessory structures include the joint capsule and ligaments and meniscus [21, 22]. The contact surfaces of the adjacent bones are covered with smooth and elastic articular cartilage. It is mainly composed of chondrocytes and ECM, which ensures vibration buffer and friction reduction during movement [23]. The joint capsule is the connective tissue membrane wrapped around the joint, divided into the inner synovial layer and the outer fibrous layer. Major synovial fluid secretion provides a suitable microenvironment for intra‐articular tissue while maintaining joint stability [23, 24].

### 2.2 The Cellular and Structural Changes in OA

#### 2.2.1 Chondrocyte

The main features of OA are the destruction of articular cartilage and the reduction of chondrocytes. This pathological process accompanied by disturbances of multiple molecular mechanisms within the joint. The following several pathological processes, including apoptosis, cellular autophagy, cellular senescence, and functional abnormalities, are summarized in this review.

In the pathological process of OA, significantly increased apoptosis leads to decreased chondrocytes and disrupts the balance of cartilage ECM synthesis and degradation. Apoptosis includes intrinsic pathways induced by intracellular signals and extrinsic pathways triggered by extracellular signals, and the two apoptotic pathways are interconnected through mitochondria [25] (Figure 2B). Intracellular Deoxyribonucleic Acid (DNA) damage, endoplasmic reticulum stress, and many other stimuli can activate the mitochondrial pathway [25, 26]. This prompts the activation of proapoptotic members of the B‐cell Lymphoma‐2 (BCL‐2) family, leading to increased mitochondrial membrane permeability and the release of substances such as cytochrome *C* to activate caspase‐9. Final activation of caspase‐3 initiates apoptosis. The death receptor pathway is initiated by the binding of cell surface death receptors to the corresponding ligands, activating caspase‐8 and in turn activating downstream caspase‐3, ‐6, ‐7 and initiating apoptosis [27] (Figure 2C). The nuclear factor‐κB (NF‐κB) and mitoase‐activated protein kinase (MAPK) signaling pathways have a biphasic role in chondrocyte survival and apoptosis [28, 29]. The interaction of the Notch signaling activation with NF‐κB signaling can lead to severe osteoarthritic lesions [30]. NF‐κB can enter the nucleus and regulate the expression of target genes and inhibit chondrocyte apoptosis [31, 32, 33] (Figure 2D). The wingless/integrated (WNT) signaling pathway are involved in the regulation of chondrocyte apoptosis, such example WNT 7a suppresses nitric oxide (NO)‐induced apoptosis, whereas TCF4 induces matrix metalloproteinases (MMPs) expression and apoptosis [25]. Multiple molecular signals in OA can induce chondrocyte apoptosis, and therapies targeting apoptotic targets in articular cartilage may be the direction of future treatment of OA.

**FIGURE 3:**
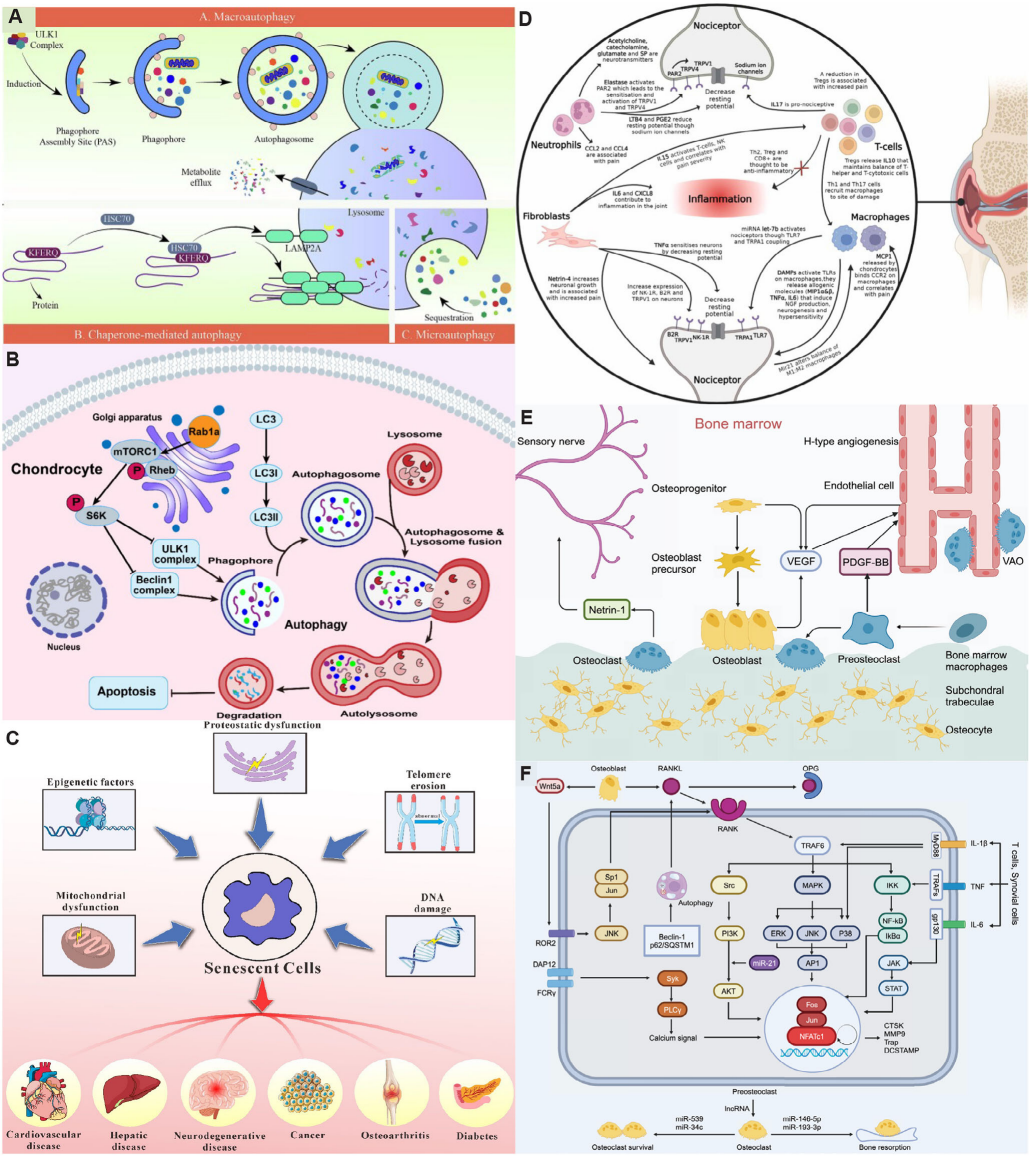
Autophagy mechanisms in chondrocytes, synovial tissue inflammation, and abnormally activated subchondral bone remodeling during osteoarthritis progression. (A) Cellular autophagy includes macroautophagy, chaperone‐mediated autophagy, and microautophagy. Reproduced with permission from Ref. [38], Copyright 2023, Elsevier. (B) An increase in Rab1a can activate the mTORC1–S6K signaling pathway to inhibit autophagy and enhance apoptosis. Reproduced with permission from Ref. [40], Copyright 2024, Elsevier. (C) The inducing factors of cellular senescence and the diseases caused by a large number of senescent cells. Reproduced with permission from Ref. [51], Copyright 2024, The authors. (D) The association between the inflammatory response resulting from the crosstalk among multiple cell types in the synovium and joint pain in OA. Reproduced with permission from Ref. [65], Copyright 2024, Elsevier. (E) In the bone microenvironment, osteoblasts secrete vascular endothelial growth factor to promote angiogenesis, while osteoclasts secrete Netrin‐1, which acts on sensory nerves and leads to joint pain. Reproduced with permission from Ref. [78], Copyright 2024, The authors. (F) Mechanistic crosstalk of subchondral osteoclasts in OA. The TRAF6 gene in the RANK/ nuclear factor‐κB ligand (RANKL)/osteoprotegerin (OPG) axis is the central factor that triggers the activation of a series of transcription factors, including NF‐κB, Protein Kinase B (AKT), MAPK, and NFATc1. Reproduced with permission from Ref. [78], Copyright 2024, The authors.

Cell autophagy is the degradation of its own components by cells through lysosomes, and normal cell autophagy is conducive to the removal of intracellular senescence and damaged organelles [34]. However, the cellular autophagy function is abnormal during OA, leading to the accumulation of damaged substances affecting the survival of chondrocytes to progress to cartilage degeneration [35]. There are many signaling pathways and molecular mechanisms regulating cellular autophagic activity, among which rapamycin kinase (mTOR) is a key protein of cellular autophagy [36, 37, 38] (Figures 2E and 3A). During cellular stress, mTORC1 activity is inhibited and separated from uncoordinated 51‐like kinase 1 (ULK1), and the ULK1 complex is activated by autophosphorylation along with activation of Atg proteins such as Atg13 to initiate cellular autophagy [39, 40] (Figure 3B). In addition, phosphorylation of adenylate‐activated protein kinase can regulate FoxO1 activity [41]; FoxO1 directly interacts with Atg proteins to control autophagosome formation and fusion with lysosomes, promoting the cellular autophagy [42]. Numerous studies have shown that the activation of cellular autophagy can effectively reduce the occurrence and development of OA [37, 43, 44]. Seeking rational increased cellular autophagy activity is significant for new therapies for OA.

Chronic age‐related pathological processes such as OA, Alzheimer's disease, and sarcopenia share the same molecular and cellular mechanisms, all belonging to low‐grade chronic systemic inflammation [45, 46]. Aging has long been recognized as an important cause of OA [47], the characteristics of cellular senescence, include genomic instability, telomere wear, epigenetic alterations, mitochondrial dysfunction, and stem cell failure [48, 49], has a huge impact on cartilage integrity and functionality [50]. The senescence‐related secretory phenotype (SASP) factors secreted by senescent cells are highly correlated with proinflammatory cytokines, chemokines, and matrix‐degrading enzymes in OA [51] (Figure 3C). SASP is also involved in the overproduction of reactive oxygen species (ROS) and activating p38 MAPK signaling, leading to DNA damage and oxidative stress. Studies have shown that the removal of senescent cytolytic agents, such as quercetin and dasatinib, can reduce the symptoms of experimental OA [52]. Cao found that exosomes of umbilical cord mesenchymal stem cells (MSCs) restored the viability of aged chondrocytes, and the p53 signaling pathway in miRNA of MSCs had a key role in alleviating cellular senescence [53]. Meanwhile, the detection of cellular senescence using the fluorescent compound C12FDG could contribute to the understanding of OA progression [54]. This provides a new direction for the treatment of OA and is expected to improve OA condition by intervening in the aging process of cells.

In OA, the chondrocytes have a decreased ability to synthesize type II collagen and proteoglycans [55]. And chondrocytes secrete a variety of metallomatrix enzymes, such as MMP‐2, MMP‐9, MMP‐13, as well as cystinase and hyaluronidase [56]. Hyaluronidase can degrade the HA in the synovial fluid [57], which reduces its lubrication and barrier effects, leading to an accelerated degradation of the cartilage matrix. Meanwhile, chondrocyte proliferation and differentiation are severely affected. Cell proliferation signaling pathways such as MAPK and PI3K/Akt are inhibited [58]. Cell cycle arrest and limits chondrocyte proliferation [59]. Part of the chondrocytes transformed to a fibrotic [60] and hypertrophic phenotype [61], secreting large amounts of collagen type X. This is associated with cartilage calcification and ossification, and the pathological calcification of cartilage further activates inflammation and matrix breakdown [1]. Moreover, when chondrocytes are subjected to abnormal mechanical loading, the internal cytoskeleton is altered, affecting cell shape and function [62]. The abnormal mechanical loading can also activate inflammatory mediators or cell apoptosis [63], such as the NF‐κB signaling pathway, resulting in a vicious cycle of cartilage degeneration. Therefore, maintaining the normal function of chondrocytes is still a non‐negligible issue in OA treatment.

#### 2.2.2 Synovial Tissue

In OA, synovial tissue mainly shows synovial hyperplasia, subintimal fibrosis, and interstitial vascularization [64]. Numerous inflammatory cells of the synovial tissue appear, including macrophages, fibroblast‐like synovial cells (FLS), neutrophils, and mast cells [65] (Figure 3D). Macrophages play a central role in synovial inflammation [66], in which a subset of CD14+ CD16+ macrophages expressed the mature macrophage marker 25F9 [67]. Proinflammatory macrophages secrete MMPs, aggregoglycanases and cyclooxygenases (cox), and also the proinflammatory factors interleukin (IL)‐6 and tumor necrosis factor (TNF)‐α [68]. Altered FLS function results in decreased lubricant and HA concentrations in the synovial fluid [69]. It can be seen that the increased inflammatory effects and decreased lubrication in the synovial tissue, leading to increased cartilage degeneration.

#### 2.2.3 Subchondral Bone

Abnormalities in the subchondral bone have dramatic effects in OA pathology [70], mainly manifested by bone sclerosis and osteophyte formation [71]. Subchondral bone thickening usually causes microfractures due to abnormal mechanical stress [72], initiating cellular and molecular responses [73]. This prompted osteoclasts to absorb old bone and osteoblasts to form new bone [74]. Osteoblasts induce the secretion of MMP‐13 and reduce proteoglycan production by chondrocytes [75], which showed abnormal expression of osteoprotegerin (OPG) and nuclear factor‐κB ligand (RANKL) [76], affecting the metabolism of the subchondral bone. Osteoclasts penetrate the cartilage junction [77], invading blood vessels and nerves deep into the cartilage and aggravating cartilage damage [78] (Figure 3E,F). Meanwhile, the number of B cells and CD68+ macrophages was significantly increased in the subchondral bone sclerosis region [79]. IL‐10 secreted by B cells leads to delayed fracture healing [80], may be involved in the recovery defect of the subchondral bone. Together, these changes drive the development of OA.

## 3 Molecular Mechanisms of OA

The progression of OA is associated with various molecular mechanisms, including alterations in cellular signaling pathways, changes in cytokine profiles, and increased oxidative stress. Within the signaling pathways, activation or suppression of pathways such as Wnt, NF‐κB, and AMPK affects processes such as cell proliferation, differentiation, and regulation. Changes in cytokines involve the modulation of the microenvironment by proinflammatory and anti‐inflammatory factors, leading to an imbalance in protective and damaging effects on the joint. Increased oxidative stress leads to intracellular redox imbalance, activates relevant signaling pathways, and further promotes inflammatory factors. Additionally, abnormal expression of proteases severely disrupts the homeostasis of the ECM. These molecular mechanisms interact and crosstalk, forming a complex regulatory network that influences the progression of OA.

### 3.1 Pathogenic Signaling Pathways

#### 3.1.1 Wnt Pathway

**FIGURE 4:**
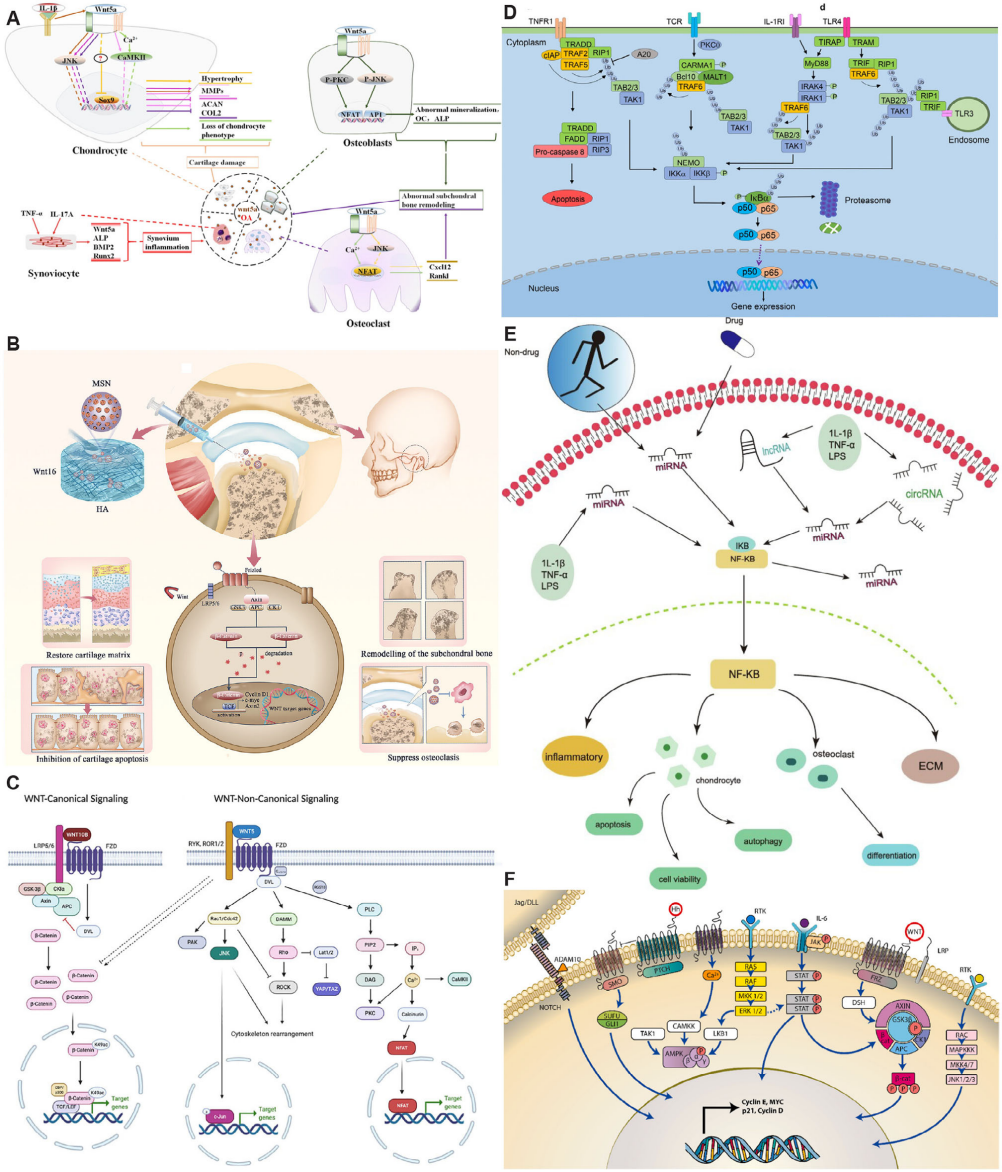
Three signaling pathways in the progression of osteoarthritis: Wnt, NF‐κB, and AMPK signaling pathways. (A) The role of cell‐specific noncanonical Wnt signaling, which is mainly activated by Wnt5a, regulates the metabolism of chondrocytes, synovial cells, osteoblasts, and osteoclasts and is involved in the development of OA. Reproduced with permission from Ref. [81], Copyright 2019, Springer Nature. (B) Schematic illustration of application and mechanism of the HA/Wnt16@MSN system for synergistic therapy in temporomandibular joint osteoarthritis by regulating Wnt/βcatenin signaling. Reproduced with permission from Ref. [89], Copyright 2024, Wiley. (C) Wingless/integrated (WNTs) can signal through β‐catenin‐dependent (or canonical) WNT signaling, WNT–PCP signaling or WNT–Ca^2+^ signaling. Reproduced with permission from Ref. [92], Copyright 2021, The authors. (D) Activation and regulation of canonical NF‐κB pathway. Reproduced with permission from Ref. [94], Copyright 2020, Springer Nature. (E) MiRNAs, lncRNAs, and circRNAs regulate the NF‐κB pathway, which influences inflammation, the ECM, chondrocyte function and osteoclast differentiation. Reproduced with permission from Ref. [96], Copyright 2022, Elsevier. (F) Most common signaling pathways involved in OA and the related crosstalk. Reproduced with permission from Ref. [102], Copyright 2024, Elsevier.

Wnt signaling involves 19 Wnt genes and multiple Wnt receptors that regulate both the classical β‐catenin dependent and nonclassical β‐catenin independent signaling pathways [81] (Figure 4A). Wnt ligands in the classical pathway bind to Frizzled receptors and recruit low‐density lipoprotein receptor‐associated protein 5/6, eventually leading to the accumulation of β‐catenin and transfer into the nucleus [82]. After transfer to the nucleus, it interacts with T cell factor/lymphatic enhanced binding factor‐1 to activate the transcription of MMP and cox. Numerous studies have demonstrated the pathological role of wnt signaling in OA and its value as a potential therapeutic target [83]. Hyperactivation of β‐catenin would accelerate the abnormal proliferation and terminal differentiation of chondrocytes through the activation of its downstream target genes c‐Myc and cyclin D1 [84]. And that in the magnesium‐deficient state, wnt/β‐catenin signaling activation reduces cellular autophagy‐mediated OA exacerbations [85]. WNT 16 has an important role in bone homeostasis and OA, including mitigation of OA, inhibition of bone resorption, and promotion of new bone formation [86]. Wnt16 can protect lumbar facet joint chondrocytes from small joint OA through the Wnt/β‐catenin pathway in patients with low back pain [87]. Wnt16 can also defend against IL‐1 β‐catenin‐induced inflammation by upregulating Lubricin and SOX9 expression and regulating RUNX2/MMP13 through the classical WNT/β‐catenin and nonclassical WNT/c‐Jun N‐terminal kinase (JNK)–cJUN signaling pathway [88]. Zhu et al. [89] fabricated mesoporous silica nanoparticles (MSNs) encapsulated by HA hydrogel for the long‐term sustained release of Wnt16 small molecules. This system has excellent chondrolosile matrix repair and ability to inhibit osteoclastogenesis, while inhibiting the overactivation of the Wnt/β‐catenin pathway (Figure 4B). In the nonclassical Wnt pathway, Wnt5a can activate JNK, p38, and Akt, and promote the expression of MMPs (MMP1, MMP3, and MMP13) [90]. Meanwhile, Wnt 5a can stimulate Leucine‐rich G‐coupled protein receptor 5 expression, and inhibition of Wnt 5a expression could partially correct the abnormal mineralization, osteocalcin secretion, and alkaline phosphatase activity of osteoblasts in OA [91]. WNT 5b inhibit chondrocyte differentiation and ECM secretion and increase the secretion of collagen I to promote fibrosis and joint degeneration [92] (Figure 4C).

#### 3.1.2 NF‐κB Pathway

NF‐κB is an important transcription factor in the cell, including five members of RelA (p65), RelB, c‐Rel, NF‐κB1 (p50), and NF‐κB2 (p52), forming homodimers and heterodimers [93]. There is a 300‐amino acid region at the N terminus of these five subunits, the v‐rel avian reticuloendotheliosis viral oncogene homolog (REL) homeodomain (RHD). The RHD mediates the dimerization of the subunits to the nucleus and binds to the corresponding DNA locus to regulate the transcription of the target genes. Normally, the NF‐κB dimer binds to the NF‐κB inhibitory protein (IκB) in the cytoplasm [94] (Figure 4D). When the cells are stimulated externally, IκB kinase (IKK) is activated and phosphorylates IκB protein. The NF‐κB dimer is then released into the nucleus to bind to the promoter region of the target gene and regulate the proliferation, immune and inflammatory responses of the cells [95]. IKK, is an enzyme complex composed of IKKα (IKK1), IKK β (IKK2), and a regulatory subunit, the NF‐κB essential regulator. It is primarily responsible for the phosphorylation of IκB [95]. IKKβ is the major subunit responsible for the phosphorylation of IκB, and it activates the classical NF‐κB pathway to participate in the rapid inflammatory response. IKKα activates the nonclassical NF‐κB pathway, which is involved in immune‐related responses. It promotes the processing of p100 to generate p52, and p52 forms a dimer with Rel B to activate target genes [93]. During the process of OA, the abnormal activation of the classical NF‐κB signaling pathway leads to an increase in p53 within the cell nucleus, which promotes the overexpression of genes such as IL‐1β, COX‐2, and prostaglandin E2 (PGE2) [96] (Figure 4E). These cytokines induce apoptosis and ECM degradation in chondrocytes. As a key subunit of the NF‐κB signaling pathway, RelA/p65 protects chondrocytes from apoptosis by inducing antiapoptotic genes, exerting a dual regulatory effect on chondrocytes [97]. Synovial inflammation also plays a crucial role in the OA microenvironment, particularly the phenotypic changes between anti‐inflammatory and proinflammatory states of synovial macrophages. Nrf2 is a transcription factor that encodes a large number of antioxidant enzymes and has antioxidant and anti‐inflammatory effects. Nrf2 reduces the intracellular ROS level by regulating the expression of antioxidant enzymes, inhibits the NF‐κB‐mediated inflammatory response, and can promote the polarization of macrophages toward the M2 phenotype [98]. Targeted therapies directed at the NF‐κB signaling pathway represent a potentially promising treatment strategy.

#### 3.1.3 AMPK Pathway

Adenosine monophosphate (AMP)‐activated protein kinase (AMPK) is a highly evolutionarily conserved cellular energy regulator. It consists of a heterotrimeric structure with a catalytic subunit α and regulatory subunits β and γ [99]. Intracellularly, AMPK mainly senses energy through changes in the ratios of AMP, adenosine diphosphate, and adenosine triphosphate (ATP), regulating physiological processes such as cellular energy metabolism and energy homeostasis [100]. When the level of AMP increases or the content of ATP decreases, phosphorylation at Thr172 triggers a conformational change in AMPK. The activation of AMPK inhibits the synthesis of lipids and proteins to reduce ATP consumption [101]. Meanwhile, it enhances catabolism to promote ATP production. AMPK deficiency in chondrocytes accelerated the progression of instability‐induced and ageing‐associated OA [100]. The AMPK signaling pathway is of great significance for maintaining the homeostasis of chondrocyte metabolism [102, 103] (Figure 4F). Liver kinase B1 (LKB1) is a crucial upstream activating kinase of AMPK. The linear ubiquitination of LKB1 promotes the activation of the AMPK pathway, suppressing the NLRP3‐mediated inflammatory response and pyroptosis in chondrocytes [104]. Moreover, the AMPK pathway has been proven to be able to inhibit the NLRP3 inflammasome and pyroptosis in OA synovial cells [105]. Meanwhile, the activation of the AMPK signaling pathway inhibits the nuclear translocation and transcriptional activity of the NF‐κB signal by regulating molecules such as SIRT1 and FOXO3 [106], which can reduce the inflammatory response in articular cartilage [107]. Additionally, AMPK can regulate peroxisome proliferator‐activated receptor γ coactivator 1α (peroxisome proliferator‐activated receptor gamma coactivator [PGC]‐1α), thereby restoring mitochondrial biogenesis and enhancing the respiratory capacity of chondrocytes [101]. In Sun's research [108], the overexpression of Sesn2 was used to activate the AMPK/PGC‐1α‐mediated mitochondrial function. This improved the intracellular mitochondrial respiratory function and effectively attenuated OA‐induced neuroinflammation. AMPK signaling collaborates with multiple signaling pathways to jointly regulate cellular behaviors. During the OA, the mTOR signaling is abnormally activated, leading to increased apoptosis and reduced autophagy. A large number of studies have utilized activated AMPK signaling to inhibit the autophagy suppressing effect of mTOR signaling [37, 109, 110], which can clear damaged organelles within cells to maintain the normal functions of chondrocytes. The activation of AMPK signaling can also inhibit β‐catenin signaling in chondrocytes to slow down the development of OA [111]. Metformin, quercetin, berberine, and the like have shown certain potential in the treatment of OA by activating the AMPK signaling pathway. However, targeted drugs with specificity and high efficiency may still be needed [112, 113, 114].

### 3.2 Cytokine

**FIGURE 5:**
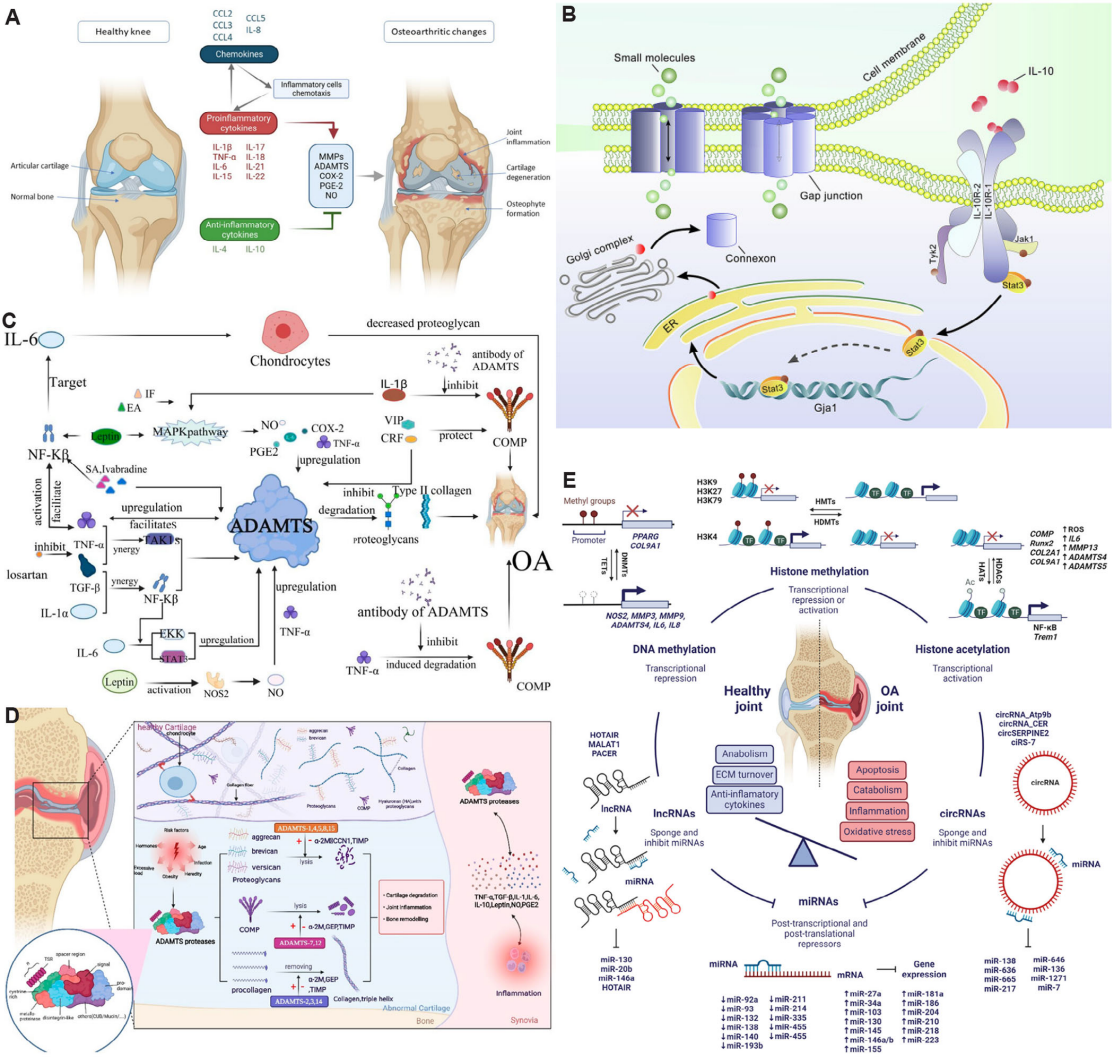
Mechanisms of key cytokines and proteases in the progression of osteoarthritis. (A) Schematic representation of key inflammatory processes and factors in osteoarthritis pathogenesis. The imbalance between proinflammatory and anti‐inflammatory cytokines, the secretion of proteases, and chemokines create a vicious cycle in osteoarthritis. Reproduced with permission from Ref. [124], Copyright 2021, The authors. (B) The schematic diagram elucidates the mechanism of IL‐10‐mediated cell‐to‐cell communication in chondrocytes. In general, IL‐10 signal enters chondrocytes through IL‐10 receptor and activates the STAT3 signaling, which finally upregulates Cx43 expression and facilitates gap junction formation between chondrocytes. Reproduced with permission from Ref. [147], Copyright 2023, Elsevier. (C) The development of OA is mediated by inflammatory factors and ADAMTS. Cytokines such as TNF‐α and IL‐6 upregulate the expression of ADAMTs, and ADAMTs degrade proteoglycans and type II collagen. Reproduced with permission from Ref. [165], Copyright 2022, The authors. (D) Structure of the ADAMTS proteases includes a signal peptide, variable‐length anterior domain, metalloproteinase domain, integrin‐like domain, central thrombospondin type 1 sequence repeat motif, cysteine‐rich spacer domain, and auxiliary domain. ADAMTS proteases degrade various proteoglycans and COMP, an important noncollagen protein in the cartilage. Reproduced with permission from Ref. [165], Copyright 2022, The authors. (E) Integrative view of the most significant epigenetic mechanisms involved in the pathogenesis of OA. Reproduced with permission from Ref. [173], Copyright 2023, The authors.

Inflammatory signaling pathway plays a key role in the development of OA, with cytokines being the dominant player. Cytokines are a class of small‐molecular weight proteins mainly secreted by immune cells and play crucial roles in cell communication, immune responses, and inflammation mediation. Cytokines in OA can be mainly classified into proinflammatory cytokines and anti‐inflammatory cytokines (Figure 5A).

Proinflammatory cytokines disrupt the homeostasis of articular cartilage by altering cell metabolism, remarkably accelerating joint damage. They primarily affect the secretion of cytokines, inflammatory compounds, proteases, and other substances by cells through intracellular signaling pathways [115]. This paper mainly summarizes the following several proinflammatory factors.

Interleukin‐1β (IL‐1β) is one of the members of the IL‐1 family and is mainly produced by M1‐type macrophages [116]. The levels of IL‐1β are significantly elevated in the cartilage, synovium, and subchondral bone of patients with OA [117]. It mainly promotes the production of enzymes such as MMPs and a disintegrin and metalloproteinase with thrombospondin motifs (ADAMTs) and induces the generation of nitric oxide and PGE2 through the NF‐κB and MAPK signaling pathways [118]. Recent research indicates that IL‐1β also upregulates the PI3K/Akt pathway to activate joint inflammation. Cucurbitacin E shows a high binding affinity for the inhibitory target PI3K [114], thereby intervening and reversing IL‐1β‐induced cartilage inflammation [119]. Chrysophanol impedes IL‐1β‐induced cartilage inflammation in OA by activating SIRT6 and influencing the NF‐κB pathway [120]. Mao restored the inflammatory response caused by IL‐1β through the overexpression of miR‐320a and found that miR‐320a mainly improved OA by targeting DAZAP1 and the extracellular signal‐regulated kinase (ERK)/JNK/MAPK‐related pathways [121].

TNF‐α is a homotrimeric protein composed of 157 amino acids [122]. It is initially synthesized in the transmembrane form within the cell. Through the action of TNF‐α converting enzyme, it is converted into soluble TNF‐α, which is then released outside the cell to exert its functions [123]. This cytokine is mainly produced by activated macrophages, T lymphocytes, and natural killer cells. The binding of TNF‐α to TNF‐R1 leads to the interaction between the TnFr1‐associated death domain protein adapter protein and other adapter proteins [124]. Through a series of complex signal transduction processes, the NF‐κB signaling pathway is activated, which is involved in the occurrence and progression of OA [125]. Zhang et al. [126] detected the cytokines in plasma extracellular vesicles (EVs) of patients with OA. It was found that EV‐associated TNF‐α was pathogenic in OA. Meanwhile, TNF‐α carried in EVs could serve as a predictive marker for the progression of OA. Bi et al. [127] utilized etanercept to inhibit TNF‐α signaling and regulate fibrocartilage stem cells (FCSCs) for cartilage repair. It was found that etanercept could specifically block the NF‐κB pathway and weaken the apoptotic effect of TNF‐α on FCSCs. Meanwhile, TNF‐α can also increase the expression of cadherin 11 in FLS through the mediation of the PI3K/Akt axis [122], exacerbating synovitis and cartilage damage [128].

IL‐6 is mainly produced by macrophages, endothelial cells, and lymphoid cells. It has multiple biological functions and plays important roles in immune responses, acute‐phase reactions, and the regulation of hematopoiesis [129]. Previous studies have reported that the levels of IL‐6 in synovial fluid and serum of patients with OA are abnormally elevated [130]. IL‐6 binds to the soluble receptor (sIL‐6R) and activates the trans‐signaling pathway with proinflammatory effects through the widely expressed gp130 protein. IL‐6 binds to the membrane‐bound receptor and activates the classical signaling pathway that confers anti‐inflammatory and regenerative properties to IL‐6 [131]. Therefore, the role of IL‐6 in the process of OA depends not only on the concentration of IL‐6, but also on the concentrations of sIL‐6R and gp130, as well as the evaluation of trans‐ and classical signaling [132]. Meanwhile, the gene polymorphism of IL‐6 directly affects its expression level, which has a significant impact on the individual differences in OA susceptibility. For example, the susceptibility and protective haplotypes within the promoter region of the IL‐6 gene influence the risk of OA in some Indian populations [133]. And the rs12700386 polymorphism in the IL‐6 gene may increase the risk of OA in Chinese Han individuals [134].

Anti‐inflammatory cytokines are cytokines that inhibit at least one of the proinflammatory cytokines responsible for the occurrence and development of OA. Their functions include suppressing the effects of proinflammatory factors, downregulating the levels of proteases, inhibiting inflammation and lysis of chondrocytes, and promoting the synthesis of proteoglycans and type II collagen by cells. This article mainly summarizes the following anti‐inflammatory factors.

IL‐4 is a protein composed of 129 amino acids, and its four interconnected α‐helices are stabilized by three disulfide bonds [135]. As a cell ligand, IL‐4 is mainly a cytokine of activated T cells [136]. It can regulate a variety of immune cells, including B cells, T cells, mast cells, macrophages, and so on [124]. Numerous studies have shown that IL‐4 significantly inhibits the expression and release of proinflammatory factors and the secretion of proteases [137, 138, 139]. The study by Von Kaeppler et al. [140] found that IL‐4 prevents OA in a bone marrow and STAT6‐dependent manner. It can also maintain joint health by promoting the polarization of macrophages toward the M2 phenotype and clearing proinflammatory debris. Ko et al. [141] discovered a novel adipokine, omentin‐1, which can induce the anti‐inflammatory effect of IL‐4 and the M2 polarization of macrophages in OA synovial fibroblasts through the PI3K, ERK, and AMPK pathways.

IL‐10 is a cytokine with pleiotropic anti‐inflammatory properties. Structurally, it is similar to interferon and initiates its function by binding to the IL‐10 receptor (IL‐10R) [136]. IL‐10 is mainly released by immune cells, including monocytes, macrophages, T cells and B cells [124]. Connective tissues, including chondrocytes, can also produce a small amount of IL‐10, which exerts its function in the ECM of chondrocyte [142]. IL‐10 activates multiple intracellular pathways to promote cartilage protection, anti‐inflammation, and antiapoptosis [143, 144]. Meanwhile, it stimulates the production of type II collagen and proteoglycans and inhibits the synthesis of MMPs [145]. Barker et al. [146] found that in patients with anterior cruciate ligament injury and knee OA, the concentration of IL‐10 in the serum and the ratio of IL‐10/TNF‐α were significantly decreased. It was speculated that this might be a biomarker for the evaluation of knee OA in the future. IL‐10 binds to IL‐10R1, activates the STAT3 signaling pathway, upregulates the expression of Cx43, promotes the formation of gap junctions between cells, enhances intercellular communication, and maintains the homeostasis of cartilage [147] (Figure 5B).

### 3.3 Oxidative Stress

**FIGURE 6:**
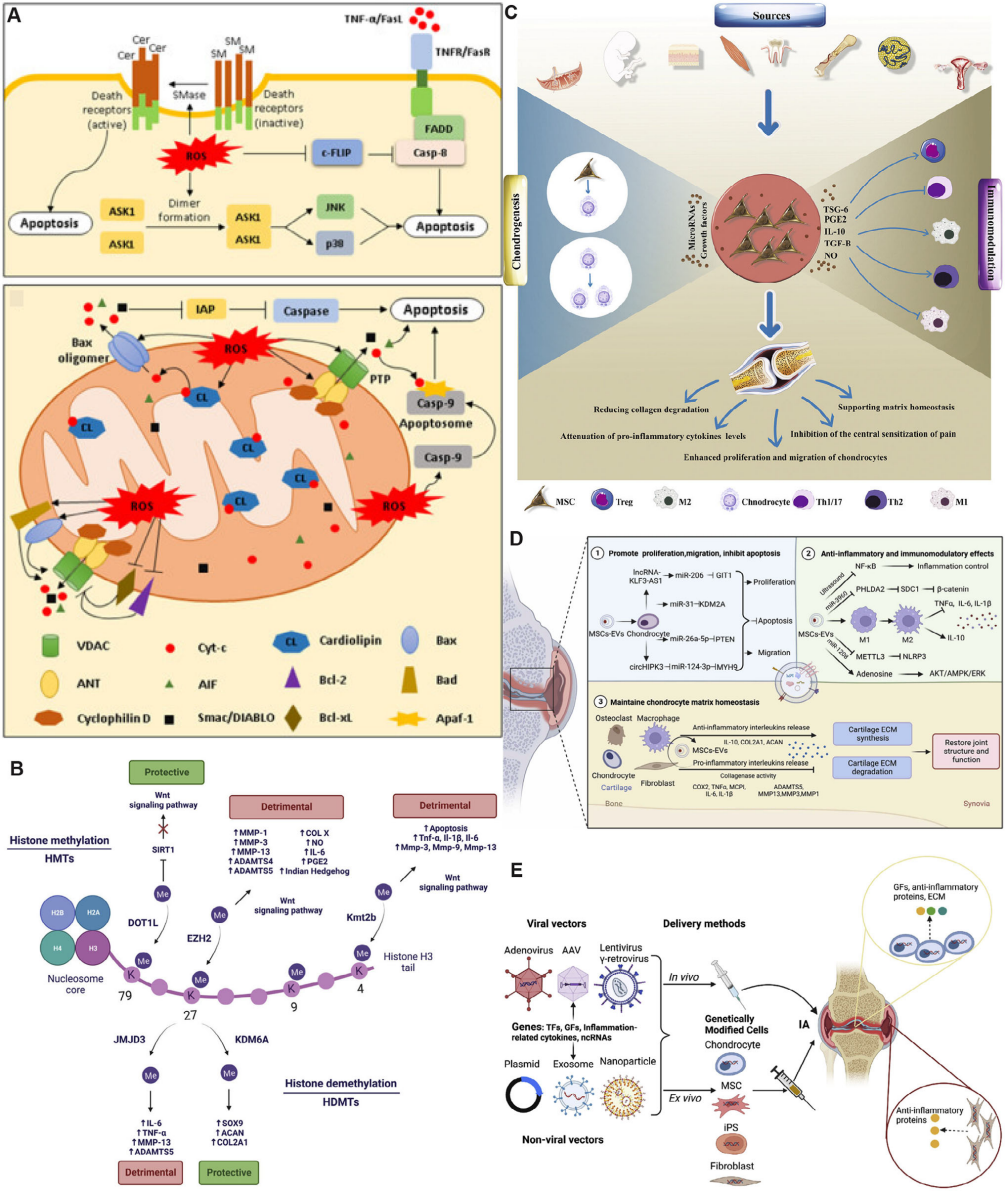
Biotherapeutic approaches in osteoarthritis: stem cell therapy, gene therapy, and EV therapy. (A) Pathways of ROS‐driven apoptotic responses include: ROS negatively regulate cellular FLICE‐inhibitory protein (c‐FLIP) by enhancing its proteasomal degradation, and ROS triggers the release of Cyt‐c through VDAC‐ and ANT‐dependent mitochondrial permeability transition. Reproduced with permission from Ref. [152], Copyright 2017, Elsevier. (B) Histone methylation and demethylation in OA. Reproduced with permission from Ref. [173], Copyright 2023, The authors. (C) The action mechanisms of Mesenchymal Stem Cells (MSCs) therapy in OA. The MSCs isolated from multiple sources elicit functional recovery in OA conditions following transplantation (mainly by intra‐articular route) through immunomodulation, differentiation to chondrocyte (partially), and enhancing chondrocyte proliferation. Reproduced with permission from Ref. [236], Copyright 2023, Wiley. (D) Modulation of MSC‐EVs for OA therapies. Different MSC‐EVs promote proliferation and migration and inhibiting the apoptosis of chondrocytes. Reproduced with permission from Ref. [244], Copyright 2023, Elsevier. (E) Overview of osteoarthritis gene therapy targets, vectors, delivery methods, and treatment processes. Commonly used genes include transcription factors, growth factors, inflammation‐associated cytokines, and noncoding RNAs. Delivery vectors consist of viral vectors (including adenovirus, adeno‐associated virus, γ‐retrovirus, and lentivirus) and non‐viral vectors (including plasmids, exosomes, nanoparticles). Reproduced with permission from Ref. [259], Copyright 2023, Oxford University Press.

Oxidative stress represents an imbalance between oxidation and antioxidation within cells, and ROS are a key inducer of oxidative stress [148]. ROS are a class of reactive molecules containing oxygen‐free radicals, mainly including superoxide anions, hydroxyl radicals, hydrogen peroxide, and so on [149]. ROS mainly originate from oxidative phosphorylation in the mitochondrial respiratory chain, NADPH oxidase, or xanthine oxidase [150]. Low levels of ROS function as intracellular redox signaling pathways and are involved in processes such as cell proliferation, differentiation, and apoptosis [151]. During oxidative stress, an excessive amount of ROS reacts with cellular macromolecules, triggering a pathological state [152] (Figure 6A). In OA, activated inflammatory cells produce a large quantity of reactive ROS through respiratory burst. ROS‐mediated damage to mitochondrial DNA (mtDNA) and lipid peroxidation lead to mitochondrial dysfunction, causing the oxidation of cardiolipin, a marker phospholipid of the mitochondrial inner membrane, and mitochondrial depolarization [153, 154]. Meanwhile, ROS activate cell cycle regulators p53 and JNK, inducing proapoptotic Bcl‐2 family proteins such as Bak and Bax in cells [155], exacerbating the permeability of the mitochondrial outer membrane and releasing cytochrome *C* and apoptosis‐inducing factor [156]. An increase in the level of ROS induces the transcription of p16, p53, and p21 through the MMK3/6‐p38 pathway or the Ataxia telangiectasia mutated (ATM)/Ataxia telangiectasia and RAD3‐related protein (ATR) pathway, halts the S phase of the cell cycle and induces cellular senescence [157]. In addition, the accumulation of ROS activates IKK, which phosphorylates IκB to release NF‐κB. NF‐κB then binds to the κB site of target genes [158], activating the NF‐κB signaling pathway. This promotes the expression of inflammatory factors, chemokines, MMPs, and so on, triggering a vicious cycle between inflammation and ROS [159].

### 3.4 Protease

Proteases play a crucial role in the occurrence and development of OA, mainly including MMPs and ADAMTs [160]. MMPs are a family of zinc‐dependent proteolytic enzymes, which are multidomain proteins with highly conserved signal peptides, propeptide domains, and catalytic domains [161]. They are mainly responsible for cleaving a variety of ECM proteins. ADAMTSs are a type of secreted zinc endopeptidases, consisting of 19 family members. They mainly contain a signal peptide, a prodomain, a metalloproteinase domain, a disintegrin‐like domain, and a central thrombospondin type 1 repeat motif [162]. In OA, the expressions of collagenases MMP‐1 and MMP‐13 are significantly increased, and MMP‐13 plays a dominant role in the degradation of the cartilage matrix [163]. MMP‐13 specifically recognizes type II collagen through its specific helical structure. The zinc ions in its catalytic domain activate water molecules to form nucleophilic hydroxyl ions, which attack the peptide bonds in type II collagen, leading to the destruction of the structure of type II collagen [56]. ADAMTS‐4 and ADAMTS‐5 are important proteases for cartilage degradation [164]. They degrade proteoglycans by acting on the interglobular domain (IGD) region of the aggrecan core protein and cleaving the Glu373‐Ala374 peptide bond [165] (Figure 5C,D). In OA, the inflammatory cytokines IL‐1β and TNF‐α produced upregulate the expressions of MMPs and ADAMTSs by activating pathways such as the MAPK pathway and the NF‐κB pathway [166].

### 3.5 Gene Regulation

Gene regulation plays a crucial role in the process of OA. Understanding the gene regulation mechanisms can contribute to revealing the pathogenesis of OA and identifying effective therapeutic targets. A large number of transcription factors are involved in gene regulation. NF‐κB is significantly activated in chondrocytes of OA, promoting the release of inflammatory factors such as TNF‐α and IL‐1β, and accelerating the process of cartilage degeneration [167]. SOX9 is a key transcription factor for maintaining the expression of type II collagen and aggrecan [168]. After the activation of the NF‐κB signaling pathway in normal chondrocytes, its member p65 binds to the SOX9 promoter to promote its expression [169]. However, during the inflammatory state of OA, the NF‐κB signaling pathway is abnormally activated. SOX9 negatively regulates its own expression at the posttranscriptional level, and these factors jointly exacerbate the deterioration of OA [170]. The activity of transcription factors may have an evaluative role in the occurrence and development of OA.

DNA methylation belongs to the mode of epigenetic modification and represents a heritable change in gene activity or function [171]. Methyl groups are transferred to the C5 position of cytosine by DNA methyltransferases (DNMTs) to form 5‐methylcytosine, thereby regulating gene expression [172]. This modification mainly occurs in CpG dinucleotide sequences or CpG islands and is associated with gene transcription repression [173]. In chondrocytes of OA, the high methylation of the PPARG promoter inhibits the expression of PPARγ, exacerbating the oxidative stress and inflammatory response in OA [174]. DNMTs downregulate the demethylation of the ADAMTS‐5 promoter, which can activate the expression of ADAMTS‐5 [175]. The IL‐6 promoter exhibits obvious hypomethylation in the OA environment, leading to its overexpression in the synovium [176]. The high methylation of the SIRT promoter reduces the binding affinity of C/EBPα, downregulates the expression of SIRT1, and then increases the acetylation level of NF‐κB p65, leading to enhanced cartilage inflammation and catabolism [177].

Histone modifications include various forms such as methylation, acetylation, phosphorylation, and ubiquitination. These modifications can alter the structure of chromatin and the accessibility of genes [178]. The state of chromatin is mainly determined by the crosstalk between DNA and histones. Regions with a high level of acetylated histones and hypomethylated DNA manifest as euchromatin, while the lack of histone acetylation and high DNA methylation are characteristics of heterochromatin [173] (Figure 5E). Histone methylation is catalyzed by histone methyltransferases and histone demethyltransferases, and it mainly occurs on lysine, arginine, and histidine residues. The most common methylation sites are on histone H3 and H4 [179] (Figure 6B). H3K9me is involved in the occurrence of temporomandibular OA, and the level of H3K9me decreases in the condylar articular cartilage of aged mice [180]. The latest research indicates that the decrease in histone H3K9 dimethylation synergizes with the DNA demethylation at the Spi‐1 binding site, which can promote the expression of ADAMTS‐5 in the articular cartilage of OA joints [181]. Assi et al. [182] demonstrated that preventing OA could be achieved by restoring the level of H3K79me through the inhibition of KDM2/7 histone demethylases. Shao et al. [183] elucidated from a genetic perspective that the overexpression of Osr2 by NSD1 methyltransferase through H3K36 methylation could improve cartilage homeostasis. Histone acetylation is catalyzed by histone acetyltransferases and histone deacetylases (HDACs). The study by Wang et al. [184] revealed that HDAC4 and HDAC8 regulate IL‐1β‐induced cartilage catabolism by modulating the MAPK/JNK and ERK signaling pathways.

Noncoding RNAs are a class of Ribonucleic Acid (RNA) molecules whose transcripts contain more than 200 nucleotides and do not encode proteins. They mainly include long noncoding RNAs (lncRNAs), circular RNAs (circRNAs), microRNAs (miRNAs), small‐interfering RNAs (siRNAs), and so on [172]. They can influence the process of OA through mechanisms such as regulating gene expression, cellular functions, and signaling pathways [185]. The lncRNA KLF3‐AS1 mediated by exosomes derived from MSCs can activate the PI3K/Akt/mTOR signaling pathway to inhibit the autophagy and apoptosis of chondrocytes [186]. The downregulation of LncRNA of a specific five‐gene set in FLS can inhibit ferroptosis and miR‐205 in FLS, and protect against inflammation and oxidative stress in the FLS of patients with OA [187]. The overexpression of lncRNA Gm37494 enhances cell proliferation, inhibits cell apoptosis and inflammation. It improves OA‐induced cartilage damage by binding to miR‐181a‐5p to increase the expression of GABRA1 [188]. miR‐132 regulates OA through the PTEN/PI3K/Protein Kinase B (AKT) signaling pathway. The overexpression of miR‐132 can promote cell proliferation and reduce chondrocyte apoptosis [189]. miRNA‐140 is specifically expressed in cartilage. MiRNA‐140‐5p inhibits chondrocyte pyroptosis and alleviates cartilage damage in OA by suppressing cathepsin B/Nod‐like receptor protein 3 [190]. During OA, hypermethylation occurs at 8 CpG sites in its upstream regulatory sequence [191], which alters the binding affinity of SMAD3, leading to a down‐regulation of miR‐140‐5p expression. Meanwhile, the inhibition of SMAD3 expression can indirectly reduce the expression of ADAMTS‐5 in OA through miRNA‐140 [192].

## 4 Diagnostic and Biomarker Development in OA

Clinical symptoms and signs in OA patients exhibit significant interindividual variability. Common imaging techniques have relatively low sensitivity for early OA diagnosis, and laboratory tests lack specific biomarkers. Current diagnostic methods are subject to numerous limitations. Therefore, identifying ideal biomarkers holds significant importance for the early diagnosis and disease assessment of OA. EVs, noncoding RNAs, and proteomic biomarkers have emerged as focal points of recent research. These novel biomarkers hold promise for offering new avenues in the early diagnosis of OA and personalized treatment strategies.

### 4.1 Limitations of the Existing Diagnostic Methods

Currently, the diagnosis of OA mainly relies on the following indicators: clinical symptoms and signs, imaging examinations, and laboratory tests [7]. Symptoms such as joint pain and joint stiffness in patients vary among different individuals. Commonly used X‐ray examinations have low sensitivity for the diagnosis of OA and it is difficult to detect soft‐tissue lesions [193]. Computed tomography can be used to observe the bone structure, but its performance in visualizing cartilage is not satisfactory [194]. Magnetic resonance imaging has a high resolution for soft tissues and is of great value for the diagnosis and assessment of early OA [195]. However, it suffers from problems such as high examination costs and long examination times [196]. Laboratory tests involve blood and synovial fluid examinations, including a mild elevation in erythrocyte sedimentation rate and C‐reactive protein. However, currently, there are no highly specific markers available for the diagnosis of OA [197].

### 4.2 Development of Biomarkers

It is particularly important to search for ideal biomarkers for the early diagnosis and assessment of the condition of OA [198]. Biomarkers including EVs, noncoding RNAs, and proteomic markers are current research hotspots [199, 200, 201]. Clarke et al. [202] used proteomics and phospholipidomics to discover the potential composite biomarkers of EVs in equine OA synovial fluid. TNF‐α carried by plasma EVs can predict the progression of knee OA [126]. Guo et al. [203] revealed that exosomal circ‐BRWD1 promotes the development of OA by regulating the miR‐1277/TRAF6 axis. Kuroiwa et al. [204] found that the methylation frequency of a specific bone morphogenetic protein 7 (BMP7) locus in the white blood cells of patients with hand OA increased significantly. It was speculated that DNA methylation might serve as a biomarker for hand OA [204]. MiRNAs play an important role in the regulation of signaling pathways such as Bax/Bcl‐2 and Wnt/β‐catenin in OA, providing potential biomarkers and therapeutic targets for the early prediction, diagnosis and treatment of OA [205]. These include: miR‐203a‐3p may be a potential candidate for staging the progression of OA [206]; miR‐126‐3p has the potential to serve as a biomarker and therapeutic target for OA [207]; EV‐mediated miR‐150‐3p serves as a potential biomarker for the diagnosis and treatment of OA [199]. Tardif et al. [208], through proteomics using mass spectrometry, discovered that CRTAC1, FBN1, VDBP, and SERPINF1 might be potential new biomarker candidates for the entire population with OA. The mRNA and protein levels of cartilage oligomeric matrix protein are elevated in OA, and it can be combined with standard diagnostic methods as an auxiliary diagnostic marker for OA [209]. Clusterin, as a molecular chaperone with cytoprotective functions, is a candidate for an early biomarker of OA [210]. The level of serum fibulin‐3 is positively correlated with the severity of OA [211], which is helpful for the diagnostic assessment of the disease. In addition, IL [212], C‐terminal telopeptide of type II collagen [213], MMPs [56], and so on are also helpful for evaluating the condition of OA and deserve our further attention.

## 5 The Treatment of OA

Current treatment approaches for OA primarily include conservative management and surgical interventions. However, conservative treatments often have limited efficacy and may carry potential side effects, while surgical treatments are associated with surgical risks and age‐related constraints. Therefore, emerging therapeutic strategies for OA are of paramount importance. These include biologics that enable precise targeting of key molecules for intervention, biotherapeutic approaches utilizing stem cells and EVs to promote cartilage repair and inflammation regulation, intelligent drug delivery systems for targeted and controlled drug release, and tissue‐engineered cartilage that constructs bioactive tissue substitutes to repair damaged joint cartilage. These emerging therapeutic approaches hold significant promise for the treatment of OA. However, challenges remain regarding their long‐term safety, large‐scale production, and clinical application.

### 5.1 Current Therapeutic Approaches

The treatment of OA mainly consists of conservative treatment and surgical treatment [197]. Among them, drug therapy is the main method of conservative treatment. NSAIDs are helpful for relieving pain but have gastrointestinal side effects, and the efficacy of chondroprotective drugs varies greatly among individuals [214]. Meanwhile, physical therapies including heat therapy and exercise can improve joint function to some extent [7]. However, for patients with severe conditions, surgical treatments are usually adopted, including arthroscopy and joint replacement [215]. Although surgical treatments can significantly improve pain and joint function, they also have drawbacks such as limitations on the age of surgery and surgical risks [216].

### 5.2 Emerging Therapeutic Strategies

#### 5.2.1 Biological Agents

With the deepening understanding of the pathogenesis of OA, especially the crucial roles of inflammatory mediators, cytokines, and growth factors therein, the treatment with biological agents has gradually become a research hotspot. Biological agents can specifically target key molecules in the pathophysiological process of OA, holding promise to provide more precise and effective treatment for OA patients.

Among the anticytokine biological agents, IL‐1 antagonists and TNF‐α antagonists are inhibitors of key inflammatory factors in the pathogenesis of OA. A large number of studies have regulated signaling pathways such as PI3K/Akt and Nrf2/NF‐κB through anti‐inflammatory and antioxidant drugs [120, 217, 218] to reduce the levels of IL‐1 and TNF‐α for the treatment of OA [219, 220, 221]. IL‐1Ra can inhibit the IL‐1 signaling pathway, reduce joint inflammation, and thus protect cartilage. In OA models of rats and rabbits, the delivery of IL‐1ra using adeno‐associated virus (AAV) [221, 222] can effectively alleviate cartilage wear and improve joint inflammation. When IL‐1ra is used in combination with SOX9 [223], it can more significantly inhibit inflammation and promote the synthesis of the ECM of chondrocytes. Zheng et al. [224] linked a TNF‐α targeting peptide to the receptor‐binding domain (RBD) of α2‐macroglobulin to prepare α2‐macroglobulin (SM). SM can interact with the LRP1 receptor on the surface of macrophages through the RBD, promoting the uptake of TNF‐α by macrophages and its transportation to lysosomes for degradation. AZ‐628, a pan‐Raf inhibitor, can inhibit TNF‐α‐induced necroptosis of chondrocytes and regulate osteoclast formation through the NF‐κB signaling pathway, thereby delaying the progression of OA [225]. Chiu et al. [226] found that the Janus Kinase (JAK) inhibitor tofacitinib inhibits the JAK1/STAT/IL6/TNFα inflammatory signaling pathway by upregulating miR‐149‐5p, promotes the activation of chondrocytes, and reduces synovitis. miR‐149‐5p may be a potential biomarker.

Growth factor based biological agents include platelet‐rich plasma (PRP) and various growth factors. Studies have used autologous PRP injection for the treatment of OA. After 2 months of treatment, it can significantly reduce the levels of IL‐1β and TNF‐α and improve the motor function of the knee joint [227] (Table 1). Moreover, PRP rich in white blood cells has a stronger expression of anti‐inflammatory mediators and is beneficial to patients with OA in the long term [228] (Table 1). However, some studies have also reported that the presence of white blood cells does not affect the clinical outcomes of PRP injection [229]. Some studies have also pointed out that for patients with mild‐to‐moderate OA, intra‐articular injection of PRP or normal saline does not lead to differences in symptoms and joint structure after the attempt [230] (Table 1). Fibroblast Growth Factor (FGF)‐18 is an important growth factor for cartilage development and repair, and its analogs can promote the repair of damaged cartilage. After a 5‐year follow‐up analysis, Eckstein et al. [231] showed that sprifermin could maintain long‐term structural changes in articular cartilage 3.5 years after treatment (Table 1). In the high‐dose group (100  g q6mo), sprifermin led to a difference in the increase of the cartilage thickness of the tibiofemoral joint by 0.05 mm, which persisted until the fifth year. Moreover, this drug had a better therapeutic response in patients with low expression of type II collagen [232] (Table 1). It may be a potential drug that can relieve symptoms and inhibit disease progression. Nerve growth factor (NGF) inhibitors can significantly improve chronic musculoskeletal pain and function. Anti‐NGF antibodies such as tanezumab, fulranumab, and fasinumab can notably improve pain and function in patients with OA. Compared with the placebo, tanezumab has statistically significant improvements in scores assessing pain and physical function [233]. However, some studies have also reported that after treatment with sprifermin, although there was an improvement in the WOMAC score, it was not statistically significant. Additionally, the application of anti‐NGF antibodies may lead to adverse reactions such as peripheral paresthesia [234]. It seems that more research is needed to determine the optimal dose and the overall risk benefit ratio [235].

**TABLE 1:** Clinical trials of OA treatment.

Therapy	Trial design	Outcomes	Conclusion	Year, author, references
Autologous PRP injection vs. etoricoxib treatment	150 knee KOA patients were randomly divided into a control group (treated with etoricoxib) and an observation group (treated with etoricoxib plus PRP injection).	The total effective rate of the observation group (94.67%) was higher than that of the control group (84.00%). The levels of related biomarkers in the observation group were lower, and the knee joint and motor function scores were higher than those in the control group.	Autologous PRP injection therapy for KOA can improve the levels of related biomarkers, can effectively enhance knee joint and motor functions, and has good clinical efficacy.	2024, Qiao [227]
Intra‐articular injection of allogeneic ADSCs vs. placebo (normal saline) treatment	40 knee OA patients were divided into two groups to receive ASCs injection and placebo injection respectively. Clinical symptoms, serum biomarkers, cell surface marker changes, and MRI changes of articular cartilage were evaluated within 1 year.	The clinical symptoms of the ADSCs group improved. The levels of HA and cartilage oligomeric matrix protein in serum decreased, the levels of inflammatory markers decreased, the expressions of CD3, CD4, and CD8 decreased, the number of CD25⁺ cells increased, and MRI showed an increasing trend in the thickness of tibial and femoral articular cartilages.	Intra‐articular injection of ADSCs is safe and effective for the treatment of knee OA, which can promote articular cartilage regeneration and symptom improvement.	2023, Sadri [238]
Intra‐articular injection of PRP vs. placebo treatment	288 patients aged 50 years and above with symptomatic mild to moderate radiographic medial knee OA were randomly assigned to receive PRP or placebo injections. The changes in pain and joint structure were evaluated at 12 months.	At 12 months, there were no significant differences in knee pain scores and changes in medial tibial cartilage volume between the PRP group and the placebo group.	In patients with symptomatic mild to moderate radiographic knee OA, intra‐articular injection of PRP had no significant improvement in symptoms and joint structure at 12 months, and is not supported for the treatment of knee OA.	2021, Bennell [230]
Comparison between LR‐PRP and LP‐PRP	Cell and cytokine analyses were performed on LR‐PRP and LP‐PRP from 12 patients with mild to moderate knee OA.	The expressions of IL‐1Ra, IL‐4, IL‐8, and MMP‐9 in LR‐PRP were significantly higher than those in LP‐PRP. There were no significant differences in nociceptive pain mediators NGF and TRAP5 between the two.	LR‐PRP is more anti‐inflammatory than LP‐PRP but may be more chondrotoxic.	2023, Jayaram [228]
Intra‐articular injection of sprifermin vs. placebo treatment	549 knee OA patients were randomly assigned to receive different doses of sprifermin or placebo injections for 18 months, and were followed up for 5 years to evaluate joint structure and symptom changes.	High dose sprifermin (100 µg every 6 months) increased the thickness of the total femorotibial joint cartilage, and the difference from the placebo persisted until 5 years. The WOMAC pain scores of all treatment groups improved by about 50%. There was no replacement of the treated knee in the high dose sprifermin group within 5 years.	Sprifermin can maintain long‐term improvement of articular cartilage structure. There may be clinical benefits in the subgroup at risk of progression, and it has good safety.	2021, Eckstein [231]
Analysis of the treatment effect of sprifermin based on PRO‐C2 levels	The levels of PRO‐C2 in serum and synovial fluid of patients in the FORWARD study were measured, and the responses of patients with different PRO‐C2 levels to sprifermin treatment were analyzed.	The level of PRO‐C2 in synovial fluid increased during sprifermin treatment. Patients with low serum PRO‐C2 levels had more cartilage loss in the placebo group and better responses to sprifermin treatment.	Patients with low serum PRO‐C2 levels have faster cartilage loss and more obvious responses to sprifermin treatment. PRO‐C2 can be used to identify patients who may benefit from treatment.	2022, Bay‐Jensen [232]
Intra‐articular injection of BMSCs vs. placebo treatment	146 patients with grade 2–3 knee OA were randomly assigned to receive BMSCs or placebo injections. The WOMAC total score, sub‐scores, VAS score, and MRI results were evaluated during 12‐month follow‐up.	The WOMAC total score, pain, stiffness, physical function sub‐scores, and VAS score of the BMSCs group improved significantly at 6 and 12 months. T2 mapping showed that the deep cartilage of the medial femorotibial compartment of the knee in the BMSCs group did not deteriorate, while that in the placebo group did. The changes in cartilage volume were not significant in both groups.	BMSCs are safe and effective for the treatment of grade 2–3 knee OA, which can relieve pain, improve function, and prevent the deterioration of cartilage quality.	2023, Gupta [239]
Intra‐articular injection of human BMSCs vs. placebo treatment	24 knee OA patients were randomly assigned to receive BMSCs or placebo injections. The scores of VAS, WOMAC, and KOOS and MRI T2 mapping results were evaluated at different time points after injection.	At 9 months, the improvement of WOMAC and KOOS pain scores in the MSC group was better than that in the control group. At 12 months, the increase in the mean T2 value of the medial compartment in the MSC group was less than that in the control group, and no serious adverse events were observed.	A single intra‐articular injection of allogeneic BMSCs can effectively relieve pain in knee OA patients at 9 months and prevent the progression of OA at 12 months.	2025, Lee [240]

Abbreviations: LR‐PRP, leukocyte‐rich PRP; LP‐PRP, leukocyte‐poor PRP; MSC, Mesenchymal Stem Cells; PRO‐C2, type II collagen formation; VAS, visual analog scale; WOMAC, Western Ontario and McMaster Universities Osteoarthritis Index; KOOS, Knee Injury and Osteoarthritis Outcome Score.

#### 5.2.2 Biotherapy

MSCs are common multipotent stem cells that can be obtained from tissues such as bone marrow, adipose tissue, and umbilical cord blood. MSCs possess the functions of self‐renewal, trans‐differentiation, and immunomodulation [236] (Figure 6C). They can reduce inflammation and provide a favorable microenvironment for the treatment of OA [237]. The injection of allogeneic adipose‐derived MSCs (ADSCs) for the treatment of OA significantly promoted articular cartilage regeneration and improved joint inflammation in the treatment group 3 months after the intervention [238] (Table 1). Allogeneic bone MSCs (BMSCs) have also been shown to effectively treat grade 2 and 3 OA, providing sustained relief from pain and stiffness [239] (Table 1). Furthermore, a single intra‐articular injection of allogeneic BMSCs can offer satisfactory pain relief for OA patients, suggesting a potential outpatient treatment option [240]. Moreover, studies have found that MSCs differentiated into chondrocytes may be more beneficial for the treatment of OA compared with BMSCs [241] (Table 1).

EVs are membrane‐bound vesicles with a diameter ranging from 30 to 5000 nm, secreted by various cells and capable of carrying multiple bioactive molecules [242]. They can participate in intercellular communication through paracrine signaling. Based on their size, EVs can be classified into apoptotic bodies, extracellular microvesicles, and exosomes, among others [243]. MSC‐EVs represent a valuable cell‐free therapeutic approach for OA [199], MSC‐EVs are involved in the regulation of cartilage function and exhibit anti‐inflammatory, immunomodulatory, and metabolic disorder‐alleviating properties [244] (Figure 6D). Warmink et al. [245] compared the therapeutic effects of MSCs and MSC‐EVs in mild OA and found that MSC‐EV treatment was more effective in reducing cartilage degeneration and joint pain. The reduction in joint pain is primarily achieved by acting on peripheral neurons to decrease the excitability of nerve branches [246]. Duan et al. [247] demonstrated that the therapeutic efficacy of OA treatment using EVs secreted by synovial MSCs pretreated with lipopolysaccharide was significantly enhanced. To achieve more effective therapeutic outcomes with MSC‐EVs, combining adipose‐derived MSC‐EVs with chitooligosaccharide can reverse the expression induced by cartilage damage, representing a novel cell‐free biomaterial therapeutic approach [248]. To safely and effectively deliver drugs to the site of disease, utilizing EVs derived from macrophages can effectively evade immune clearance. Additionally, incorporating targeting peptides on the surface of these EVs endows the vesicle system with low toxicity and high targeting capability [249]. This approach can effectively restore the reconstruction of subchondral bone. Furthermore, a nanovesicle system composed of MSC‐EVs loaded with siRNA and insulin‐like growth factor‐1 has been developed. This system achieves the downregulation of inflammatory factors and the upregulation of cartilage regeneration factors, while also enabling long‐term retention at the site of disease. It possesses both cartilage‐protective properties and cartilage‐adhesive capabilities [250]. However, the research methods for EVs are currently in a transitional phase. The large‐scale production processes are not yet perfected, and the pharmacokinetic properties within the body remain unclear. Additionally, long‐term safety may still require further in‐depth evaluation.

Mitochondria, serving as the “powerhouses” of the cell, play a critical role in cellular metabolism and redox balance. In OA, when chondrocytes are subjected to oxidative stress and inflammatory factors [251], the morphology of intracellular mitochondria changes, and their respiratory function and ATP synthesis decline [252]. This leads to a reduction in the synthesis of the ECM. Mitochondrial dysfunction leads to the release of mtDNA and mitochondrial ROS, which activate the NF‐κB signaling pathway [253]. Simultaneously, this process triggers the intrinsic apoptosis pathway and the caspase cascade, promoting cellular inflammation and apoptosis [254]. Therefore, the restoration of mitochondrial function holds significant importance for the treatment of OA. Mitochondria isolated from MSCs can be transferred to joint cells. These mitochondria can remain in the joint and alleviate the inflammatory response in OA [255]. EVs enriched in mitochondria derived from human synovial fluid MSCs have been shown to improve mitochondrial function in articular cartilage and reduce levels of oxidative stress and cellular senescence proteins [249]. EVs enriched in mitochondria derived from human synovial fluid MSCs have been shown to improve mitochondrial function in articular cartilage and reduce levels of oxidative stress and senescence‐associated proteins [256]. While the loss of sirtuin 3 (SIRT3), a member of the sirtuin family of Nicotinamide Adenine Dinucleotide (NAD)+‐dependent deacetylases, has been shown to promote the development of OA, SIRT3‐mediated deacetylation of cytochrome *C* oxidase subunit 4 isoform 2 can rescue the impaired mitochondrial respiratory chain function in OA, thereby improving the OA phenotype [257]. As an essential physiological metabolite of the tricarboxylic acid cycle in mitochondria, α‐ketoglutarate can reverse the effects induced by IL‐1β by promoting mitophagy and inhibiting the production of ROS [251]. Chen et al. [258] applied targeted hydrogel microspheres loaded with mitophagy activators to promote mitophagy, achieving subcellular therapy while simultaneously improving the degradation of cartilage matrix and the formation of osteophytes. These studies highlight the potential therapeutic approach of maintaining mitochondrial quantity, respiratory function, and autophagic function for OA.

Gene therapy for OA primarily involves modulating the expression of genes associated with OA pathogenesis, inhibiting inflammatory responses, and promoting cartilage repair. This strategy involves delivering genes, miRNAs, and short hairpin RNAs either individually or using carriers to target tissues [259] (Figure 6E). Common gene delivery vectors include viral vectors and nonviral vectors, such as adenoviruses, AAVs, and liposomes. The targets for gene therapy include inflammatory factors, growth factors, and transcription factors [260]. IL‐1 receptor antagonist (IL‐1ra) has demonstrated significant therapeutic effects in both rat models of OA [222, 261] and equine posttraumatic OA [262]. A single injection of AAV2–FGF18 gene therapy can reduce cartilage loss and subchondral bone damage in a mechanically induced OA model [263]. And rAAV–hFGF‐2 improves sodium iodoacetate‐induced OA in rats by inhibiting toll‐like receptor (TLR)‐4 signaling and activating TIMP‐1 [264]. Cai et al. [265] utilized nanoparticles loaded with plasmid DNA encoding transforming growth factor‐β (TGF‐β) to enhance cartilage regeneration, demonstrating superior glycosaminoglycan deposition and type II collagen expression compared with pure MSCs. The upregulation of Runx1 can enhance the homeostasis of the entire joint [266], and the injection of AAV–Runx1 has been shown to maintain better joint cartilage integrity and a higher amount of proteoglycans compared with the OA group [267]. Noncoding RNA plays a significant role in gene delivery [268]. The delivery of mRNA encoding the transcription factor Runx1 effectively promotes cartilage regeneration [269]. Additionally, recombinant FGF18 mRNA encapsulated in lipid nanoparticles can be used for targeted treatment of OA [270]. Zhang et al. [271] utilized pH‐responsive metal‐organic frameworks to deliver hypoxia‐inducible factor (HIF)‐2α siRNA, effectively improving joint cavity stability. Furthermore, a combined RNA interference therapy, incorporating thermosensitive hydrogels and siMMP13 encapsulated in liposomes, was shown to effectively inhibit cellular inflammation, apoptosis, and oxidative stress [272].

#### 5.2.3 Smart Drug Delivery Systems

**FIGURE 7:**
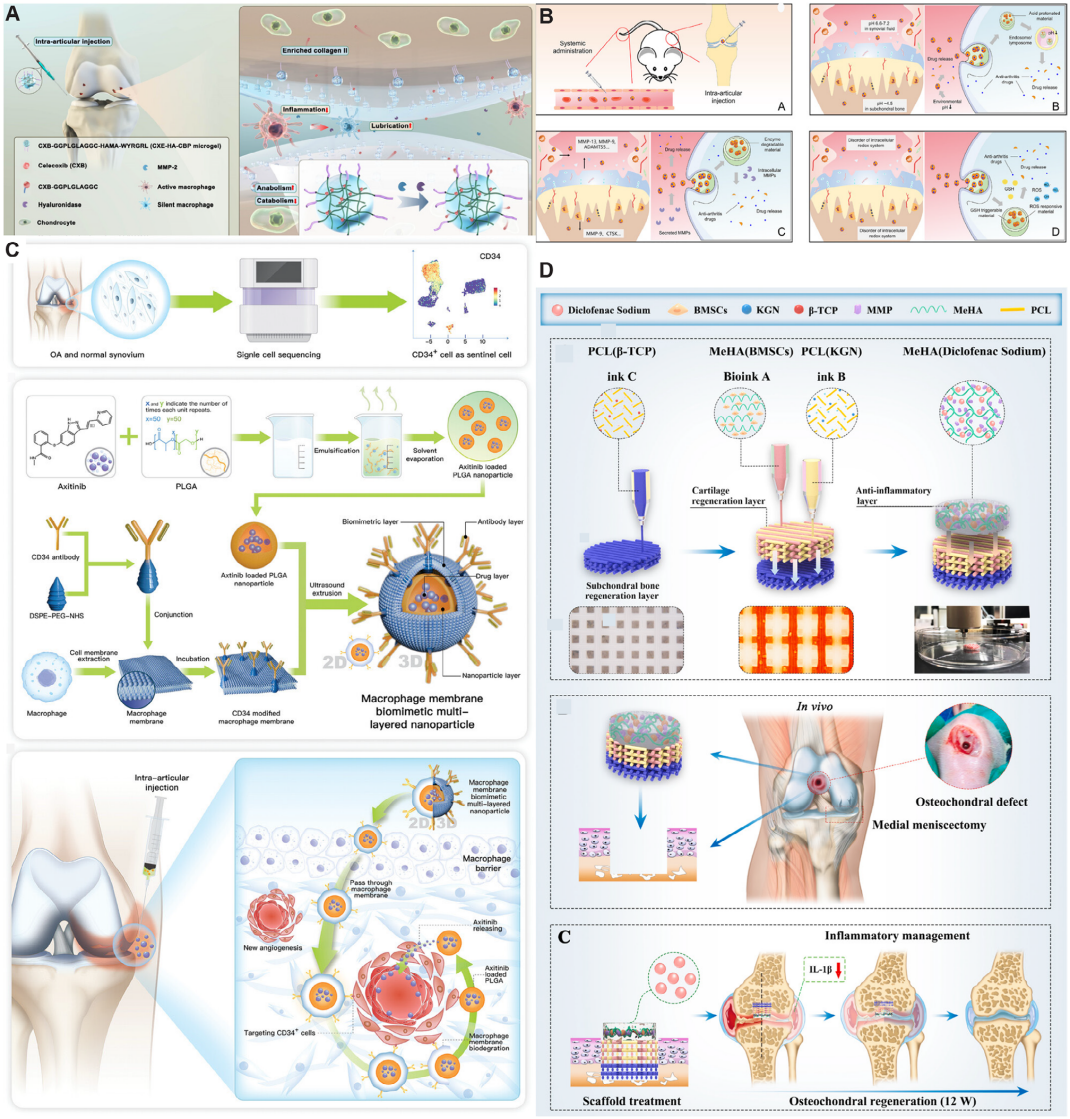
Emerging therapeutic strategies for osteoarthritis: smart drug delivery systems and tissue‐engineered cartilage. (A) In vivo osteoarthritis treatment performance of celecoxib and collagen II binding peptide modified HA based microgel via inflammation suppression, chondrocyte protection, and joint lubrication improvement. Reproduced with permission from Ref.(B) The principle of endogenous stimuli‐responsive materials releasing drugs includes: different drug administration methods, extracellular drug release in an acidic microenvironment, drug release induced by enzyme‐responsive effects, and drug release by substances involved in redox reactions. Reproduced with permission from Ref. [280], Copyright 2022, Elsevier. (C) Schematic diagram of macrophage membrane biomimetic multilayered nanoparticles alleviating the progression of OA by inhibiting synovial angiogenesis, including the targets of synovial angiogenesis, the preparation of axitinib‐loaded PLGA nanoparticles, and the preparation of macrophage membrane biomimetic multilayered nanoparticles modified with CD34 antibody. Intra‐articular injection of macrophage membrane biomimetic multilayered nanoparticles can pass through the macrophage barrier, target CD34+ cells and slowly release axitinib. Reproduced with permission from Ref. [281], Copyright 2025, Wiley. (D) Flowchart of scaffold fabrication and scaffold treatment for osteochondral defects in osteoarthritic joints. The bottom of the scaffold is composed of a β‐TCP porous scaffold to mimic the trabecular structure of the subchondral bone. The middle layer of the scaffold is composed of PCL–KGN and MeHA bioink containing BMSCs, featuring a multimaterial alternating printing pattern for cartilage regeneration. The hydrogel layer of MMP–MeHA coated on the top layer of the scaffold is used to manage the inflammatory and painful impacts of cartilage lesions. The designed scaffold was implanted into a rat model with severe joint injury for evaluation. The implantation treatment of the designed scaffold can effectively repair the osteochondral defects of the osteoarthritic joints and prevent the progression of osteoarthritis. Reproduced with permission from Ref. [295], Copyright 2021, Elsevier.

Current pharmacological treatments for OA suffer from uneven systemic drug distribution, limited therapeutic efficacy at the site of disease, and increased systemic adverse reactions [273]. Smart drug delivery systems, utilizing carriers such as nanoparticles, liposomes, and hydrogels, enable targeted drug delivery and controlled release at the joint site [274]. Primarily, these systems leverage microenvironment‐sensitive responses and targeted drug carriers to achieve effective OA treatment [275]. The joint microenvironment in patients with OA is characterized by the accumulation of acidic substances such as lactic acid within the joint cavity due to factors like inflammatory responses and oxidative stress, resulting in a lower pH compared with normal tissues [276]. Potential of Hydrogen (PH)‐responsive drug delivery systems respond to the low pH conditions within the OA joint cavity, undergoing structural degradation and releasing their biologically active components. Pan et al. [277] utilized pH/redox‐responsive nanogels to deliver geraniol, which significantly alleviated oxidative stress and inflammatory responses. He et al. [278] employed mesoporous silica nanoparticles and pH‐responsive polyacrylic acid to load and deliver andrographolide. This system enables sustained release of Andrographolide (AG) in the acidic environment of OA joints, thereby mitigating the progression of OA. Enzyme‐responsive drug carriers exploit the high expression of specific enzymes at the site of disease to trigger drug release [275]. Chen et al. [279] utilized a MMP‐2 sensitive peptide to conjugate celecoxib to methacrylate HA (gelatin methacrylate [GelMA]) microgels. During the progression of arthritis, the MMP‐2 enzyme specifically cleaves the connecting peptide, thereby releasing the drug. This approach achieves sustained drug release and maintains therapeutic concentrations (Figure 7A).

The application of targeted drug delivery mechanisms has brought new opportunities for the treatment of OA [280]. Passive targeting mechanisms leverage the increased vascular permeability caused by inflammation, allowing drug carriers to accumulate and penetrate into the diseased tissue. This enhanced permeability facilitates the retention and delivery of therapeutic agents specifically at the site of inflammation, thereby improving the efficacy and reducing off‐target effects. Active targeting, on the other hand, involves conjugating specific ligands to the surface of nanocarriers, enabling them to specifically bind to receptors on the surface of diseased cells in OA (Figure 7B). This targeted approach ensures that the therapeutic agents are delivered directly to the intended site, enhancing the treatment efficacy and minimizing side effects on healthy tissues. Liao et al. [281] developed a biomimetic nanoparticle that combines Poly (lactic‐co‐glycolic acid) (PLGA) loaded with axitinib and macrophage membranes modified with CD34 antibodies. This nanoparticle can specifically deliver axitinib to the sublining layer (SL) and inhibit angiogenesis, thereby alleviating synovial inflammation‐mediated OA (Figure 7C). Ebada et al. [282] prepared chrysophanol acid‐loaded nanoreservoirs and functionalized them with chondroitin sulfate. They compared the therapeutic effects of active cartilage targeting and passive targeting using these particles. The results showed that drug‐loaded particles functionalized with chondroitin sulfate were more effective in promoting drug retention and cartilage targeting. Additionally, a study has developed a delivery system for senolytic peptides based on magnetic nanoemulsions [283]. Utilizing the superparamagnetic properties of magnetic nanoparticles (MNPs), this system can precisely deliver the senolytic peptide drugs to the site of disease and has demonstrated excellent antiaging effects.

#### 5.2.4 Tissue Engineering Cartilage

The structure and functional specificity of articular cartilage limit the available treatment options for cartilage damage, often resulting in suboptimal therapeutic outcomes. Tissue engineering has opened new avenues for the treatment of OA, aiming to utilize principles from biology and engineering to construct bioactive tissue substitutes, thereby achieving functional repair and regeneration of joint tissues. The construction of tissue engineering primarily involves three core elements: seed cells, biomaterial scaffolds, and bioactive factors.

In the strategies for tissue engineering to repair articular cartilage, seed cells primarily focus on mature chondrocytes and MSCs. Alginate hydrogels delivering autologous nasal chondrocytes have been shown to induce excellent articular cartilage regeneration in rabbit osteochondral defects [284], with performance closely resembling that of normal cartilage in terms of both chondrogenic marker gene expression and biomechanical testing. Zheng et al. [285] isolated and cultured chondrocytes from rat rib cartilage and used these cells to construct scaffold‐free tissue‐engineered constructs. These constructs effectively repaired articular cartilage defects and showed enhanced cartilage repair efficacy following ascorbic acid treatment. Sources of MSCs include bone marrow, adipose tissue, and synovium. Karabıyık Acar et al. [286] developed a three‐dimensional (3D) scaffold using a composite of chitosan (CS) and sodium hyaluronate through complex coacervation. BMSCs were encapsulated within this scaffold. The detection of chondrogenic markers revealed that the BMSCs exhibited excellent chondrogenic differentiation. PLGA/poly (ethylene glycol) (PEG) hydrogels exhibit excellent biocompatibility with BMSCs and significantly promote the formation of new cartilage in a rat cartilage defect model [287]. Nonaka et al. [288] utilized adipose‐derived stem cells isolated from rats and bio‐3D printing to create pure cellular cartilage constructs. Through the induction of basic fibroblast growth factor (bFGF) and BMP2, these constructs were engineered to possess mechanical and histological properties similar to those of native articular cartilage. Furthermore, chondrocytes derived from induced pluripotent stem cell (iPSC)‐derived MSCs have shown significant therapeutic effects in an OA model in rabbits [289]. This therapeutic potential may be attributed to their anti‐inflammatory and anticatabolic properties, which can help salvage cartilage defects. These findings suggest that iPSC‐derived MSCs could be an emerging source of seed cells for future regenerative therapies.

Cartilage tissue engineering aims to mimic natural cartilage and restore joint function, with biological scaffolds playing a crucial role as carriers and substrates. In tissue engineering, biological scaffolds must meet several key requirements: they need to exhibit excellent biocompatibility, be capable of integrating with surrounding tissues, be customizable in terms of shape and size, and possess sufficient mechanical properties [290]. Scaffolds are primarily composed of either natural materials or synthetic materials, including collagen, silk fibroin, HA, polylactic acid, polyglycolic acid, and polyurethane [291]. To emulate the natural properties of articular cartilage, these materials are utilized to construct scaffolds. By adjusting the material composition and structure, these scaffolds can better support cell growth, differentiation, and ECM synthesis [292]. Additionally, the use of 3D printing technology to fabricate fiber scaffolds and interpenetrating network hydrogels more accurately mimics the mechanical properties of articular cartilage, providing superior mechanical support for cartilage repair. These advanced techniques enable the precise control of scaffold architecture and mechanical properties, thereby enhancing the overall efficacy of cartilage tissue engineering strategies. Li et al. utilized 3D printing to fabricate a hydrogel scaffold composed of silk fibroin and tyramine‐substituted gelatin [293]. The scaffold was designed with large pores to facilitate the transport of oxygen and nutrients, while also exhibiting excellent structural stability and mechanical properties. When combined with stem cells, this scaffold demonstrated outstanding potential for cartilage tissue repair and regeneration in a rabbit cartilage defect model. While individual natural materials may fall short in terms of manufacturing processes and mechanical performance, the incorporation of poly(ε‐caprolactone) (PCL) and PEG fibers into GelMA has been used to fabricate a multilayer scaffold. This scaffold successfully mimics the structural gradient of native osteochondral tissue, enabling simultaneous regeneration of both cartilage and subchondral bone in vivo [294]. The combination of natural and synthetic materials for therapeutic applications may be an attractive strategy. Liu et al. [295] designed a 3D bioprinted multilayered scaffold containing BMSCs. The scaffold was fabricated using MeHA/polycaprolactone, combined with kartogenin (KGN) and β‐tricalcium phosphate (β‐TCP), and was applied for the repair of osteochondral defects within each region. The BMSC‐laden MeHA was designed to actively introduce bone marrow MSCs in situ. Additionally, diclofenac sodium was induced to be incorporated into the MeHA modified with MMP‐sensitive peptides as an anti‐inflammatory strategy (Figure 7D). Li et al. [296] utilized a 3D‐printed PCL scaffold to provide fundamental mechanical support, which was combined with a CS hydrogel. This composite scaffold contains synovial MSCs (SMSCs) and recruit tetrahedral framework nucleic acid (TFNA). TFNA, which is a promising DNA nanomaterial for improving the regenerative microenvironment, could be taken up into SMSCs and promoted the proliferation and chondrogenic differentiation of SMSCs. This approach significantly enhanced the repair of cartilage defects. Furthermore, the use of stimulus‐responsive intelligent scaffolds holds potential as a therapeutic strategy for repairing cartilage defects in OA. A scaffold composed of piezoelectric conductive decellularized ECM and modified gelatin can generate electrical stimulation during joint movement. The positively charged upper layer of the scaffold attracts BMSCs and promotes their chondrogenic differentiation, while the negatively charged lower layer induces BMSCs to undergo osteogenic differentiation. This scaffold offers a promising approach for the repair of osteochondral defects [297]. Magnetic‐responsive scaffolds also show promising potential for cartilage repair [298]. By incorporating MNPs into a gelatin solution, a 3D magnetic scaffold was fabricated via electrospinning and physical crosslinking [299]. This scaffold can induce mechanical stimulation in MSCs through up‐and‐down motion under the influence of an alternating magnetic field. The citrate‐modified MNP scaffold significantly upregulates the expression of chondrogenesis‐related genes, such as COL2A1 and ACAN. Additionally, it demonstrates superior osteochondral repair capabilities in a rabbit osteochondral defect model. Gang et al. [300] utilized mild hyperthermia in tissue engineering to promote tissue regeneration. They developed a multifunctional drug‐delivery hydrogel composed of nanoscale Fe₃O₄ particles, CS, and polyolefin. This hydrogel exhibits mechanical properties that match those of articular cartilage, along with excellent magnetothermal properties and sustained drug release capabilities. In a rat model of rheumatoid arthritis, this hydrogel demonstrated significant anti‐inflammatory effects and cartilage repair. By integrating hyperthermia and chemotherapy with tissue engineering, this hydrogel scaffold offers a novel approach for the treatment of cartilage injuries.

Stimulatory factors in tissue engineered cartilage can accelerate, induce, and enhance cartilage repair, primarily through biochemical and physical stimuli. Various growth factors involved in cartilage growth and development, such as TGF‐β, insulin‐like growth factor, and BMPs, have positive effects on the chondrogenic differentiation of MSCs [301]. TGF‐β has shown promising potential in cartilage tissue engineering when delivered through various methods, including microsphere‐loaded materials [302], hydrogel systems [303], and in combination with 3D scaffolds [304]. Li et al. [305] utilized a silk fibroin hydrogel loaded with BMP‐2 and incorporated CS nanoparticles conjugated with TGF‐β. This system promoted chondrogenesis in both in vitro and in vivo models of BMSCs through the release of TGF‐β1 and BMP‐2. Another study demonstrated that the codelivery of SMSCs with TGF‐β1 and BMP‐4 effectively repaired osteochondral defects [306]. Simultaneously, given that articular cartilage resides in a hypoxic environment and is subjected to mechanical loading, low oxygen tension and mechanical stimulation also play positive roles in the construction of tissue engineered cartilage. Stem cells preconditioned under hypoxic conditions have been shown to enhance chondrogenic differentiation and osteochondral integration in articular cartilage defects [307]. Mechanical stimulation, utilizing mechanical compression, fluid shear stress, hydrostatic pressure, and osmotic pressure, activates cellular mechanosensors such as primary cilia, integrins, and ion channels. These mechanosensors regulate downstream signaling pathways that influence the proliferation and differentiation of chondrocytes [308]. Scaffolds in tissue‐engineered cartilage mimic the mechanical environment of native cartilage, providing support for cell differentiation. When combined with stretching and gallic acid, these scaffolds reduce inflammation while promoting the production of ECM by chondrocytes [309]. Appropriate compressive stimulation also significantly enhances the expression of chondrogenic markers such as COL2A1, aggrecan, and proteoglycan 4 [310].

## 6 Conclusion and Outlook

OA, as a common chronic joint disease, exhibits a complex pathogenesis involving abnormalities at multiple levels, including molecules, cells, and tissues. This review systematically explores the pathological mechanisms, molecular signaling pathways, cellular changes, and emerging therapeutic strategies of OA, unveiling a multifactorial regulatory network underlying its progression, with inflammation, metabolic dysregulation, oxidative stress, and cellular senescence playing central roles. The alterations in chondrocyte structure and function are pivotal in this pathological process. Under OA conditions, reduced autophagic activity in chondrocytes leads to the accumulation of damaged substances, affecting cell survival, while increased apoptosis results in a reduction in chondrocyte numbers. Cellular senescence and functional abnormalities further impair the synthesis capacity of the ECM. This pathological process disrupts the balance between matrix synthesis and degradation in the cartilage ECM. The hyperplasia, fibrosis, and angiogenesis of synovial tissue, along with the secretion of numerous inflammatory cells, alter the joint cavity microenvironment. These changes lead to the release of various inflammatory cytokines and matrix‐degrading enzymes, increasing inflammatory activity within the joint cavity and reducing joint lubrication efficiency. Abnormal bone resorption and remodeling in the subchondral bone affect the microstructure and mechanical properties of cartilage, further accelerating cartilage degeneration and promoting the progression of OA. At the molecular level, aberrant activation or suppression of multiple signaling pathways, including Wnt, NF‐κB, and AMPK, can directly influence the functions of chondrocytes, synoviocytes, and osteocytes. Additionally, these pathways mediate the effects of inflammatory cytokines, oxidative stress, and various proteases on the microenvironment. Elevated inflammation and ROS further promote the activation of multiple inflammatory signaling pathways and the expression of proteases, creating a vicious cycle of inflammatory milieu and cartilage degradation within the joint cavity.

Although current diagnostic methods have limitations in early detection of OA, studies on biomarkers based on exosomes, noncoding RNAs, and proteomics offer new hope for the early diagnosis and disease assessment of OA. These biomarkers not only reflect the pathological progression of OA but may also provide a basis for personalized treatment. Understanding the key aspects of OA pathogenesis, whether from the perspectives of gene regulation, signaling pathway activation, inflammatory cytokine expression, or protease production, is crucial for developing targeted diagnostics and therapeutics that target cells, critical signaling pathways, and cytokines. Although numerous studies have attempted to diagnose and treat OA early using cytokines or genetic biomarkers, the long‐term safety and efficacy of biologics and gene therapies remain to be further validated, particularly across different age groups and stages of the disease. Moreover, the formation and progression of OA are not caused by a single signal or cytokine; rather, multiple signaling pathways interact and crosstalk, leading to suboptimal efficacy when targeting individual pathways or factors alone. The large‐scale production and clinical translation of intelligent drug delivery systems still face technical challenges, and ensuring the stability and targeting of drugs within the body remains an unresolved issue, making widespread clinical application in the short term difficult to achieve. Tissue engineering technologies have shown potential in cartilage repair, but the differences between engineered cartilage and native cartilage in terms of structure and function still need to be minimized, particularly in mechanical properties and long‐term stability.

Given the variability in genetic background, lifestyle, and disease progression among OA patients, traditional one‐size‐fits‐all treatment approaches are often inadequate to meet the diverse needs of all individuals. Therefore, OA treatment should emphasize multidisciplinary collaboration and personalized care. On one hand, the integration of molecular biology, materials science, and artificial intelligence technologies could enable the development of more precise diagnostic tools and therapeutic strategies. Through techniques such as genetic testing and biomarker analysis, it may become possible to accurately subtype patients and tailor individualized treatment plans, which could represent a future trend in disease management. On the other hand, the development of combination therapy strategies is expected to emerge as a key area of research in the future. For example, combining biologics with gene therapy, or integrating tissue engineering techniques with intelligent drug delivery systems, may yield synergistic effects in cartilage repair and inflammation regulation. Such interdisciplinary approaches could address the multifaceted nature of OA, leveraging the strengths of each technology to enhance therapeutic outcomes. The development of drugs with higher specificity and targeting capabilities, designed to precisely act on key targets in the OA pathogenesis, should also consider the targeting of multiple pathways simultaneously. MSCs and EVs hold significant promise in OA treatment, as these therapeutic approaches integrate self‐renewal, immunomodulation, and differentiation capabilities. Additionally, a variety of bioactive molecules can participate in intercellular communication, further enhancing their therapeutic potential. In the future, further optimization of large‐scale preparation processes and in‐depth research on in vivo metabolism will provide a more robust foundation for their clinical application. Moreover, as our understanding of the pathological mechanisms underlying OA deepens, therapeutic strategies targeting cellular metabolism and mitochondrial function may emerge as novel breakthroughs. Regulating mitochondrial autophagy, restoring mitochondrial function, or targeting mitochondria‐associated signaling pathways could offer fresh perspectives for the treatment of OA. For the treatment of late‐stage OA, the application of tissue engineering techniques may be necessary. 3D printing technology and stimuli‐responsive smart materials can better mimic the heterogeneous structure of articular cartilage and regulate the microenvironment for cartilage regeneration. Developing more biomimetic scaffold structures and intelligent responsive materials represents a key focus of future cartilage tissue engineering research. Integrating stimulatory factors and seed cells during scaffold fabrication may also be a promising research direction.

The treatment of OA is shifting from traditional symptom alleviation to targeting pathological mechanisms and promoting tissue regeneration. Future research must establish closer connections between basic science and clinical applications to facilitate the clinical translation of more innovative therapeutic strategies. Additionally, interdisciplinary collaboration and the integration of emerging technologies will offer greater potential for OA treatment, ultimately enabling precise, personalized, and long‐term management of the condition.

## Author Contributions

Conceptualization: Bing‐Bing Xu and Jian‐Quan Wang; formal analysis: Wei Liu and Ning‐Yi Guo; investigation: Wei Liu and Ning‐Yi Guo; methodology: Wei Liu, Ning‐Yi Guo, and Bing‐Bing Xu; project administration: Bing‐Bing Xu; writing—original draft: Wei Liu and Ning‐Yi Guo; writing—review and editing: Wei Liu, Jian‐Quan Wang, and Bing‐Bing Xu. All authors have read and approved the final manuscript.

## Ethics Statement

The authors have nothing to report.

## Conflicts of Interest

The authors declare no conflicts of interest.

## Data Availability

The authors have nothing to report.
